# Lessons From Heat Stroke for Understanding Myalgic Encephalomyelitis/Chronic Fatigue Syndrome

**DOI:** 10.3389/fneur.2021.789784

**Published:** 2021-12-13

**Authors:** Dominic Stanculescu, Nuno Sepúlveda, Chin Leong Lim, Jonas Bergquist

**Affiliations:** ^1^Independent Researcher, Sint Martens Latem, Belgium; ^2^CEAUL—Centro de Estatística e Aplicações da Universidade de Lisboa, Lisbon, Portugal; ^3^Department of Mathematics and Information Science, Warsaw University of Technology, Warsaw, Poland; ^4^Lee Kong Chian School of Medicine, Nanyang Technological University, Singapore, Singapore; ^5^Analytical Chemistry and Neurochemistry, Department of Chemistry—BMC, Uppsala University, Uppsala, Sweden; ^6^The ME/CFS Collaborative Research Center at Uppsala University, Uppsala, Sweden

**Keywords:** post-viral fatigue, myalgic encephalomyelitis/chronic fatigue syndrome, gut permeability, systemic inflammatory response syndrome (SIRS), heat stroke, endotoxemia, ME/CFS, splanchnic vasoconstriction

## Abstract

We here provide an overview of the pathophysiological mechanisms during heat stroke and describe similar mechanisms found in myalgic encephalomyelitis/chronic fatigue syndrome (ME/CFS). Both conditions are characterized by disturbed homeostasis in which inflammatory pathways play a central role. Splanchnic vasoconstriction, increased gut permeability, gut-related endotoxemia, systemic inflammatory response, central nervous system dysfunction, blood coagulation disorder, endothelial-cell injury, and mitochondrial dysfunction underlie heat stroke. These mechanisms have also been documented in ME/CFS. Moreover, initial transcriptomic studies suggest that similar gene expressions are altered in both heat stroke and ME/CFS. Finally, some predisposing factors for heat stroke, such as pre-existing inflammation or infection, overlap with those for ME/CFS. Notwithstanding important differences - and despite heat stroke being an acute condition - the overlaps between heat stroke and ME/CFS suggest common pathways in the physiological responses to very different forms of stressors, which are manifested in different clinical outcomes. The human studies and animal models of heat stroke provide an explanation for the self-perpetuation of homeostatic imbalance centered around intestinal wall injury, which could also inform the understanding of ME/CFS. Moreover, the studies of novel therapeutics for heat stroke might provide new avenues for the treatment of ME/CFS. Future research should be conducted to investigate the similarities between heat stroke and ME/CFS to help identify the potential treatments for ME/CFS.

## Introduction

Heat stroke results from a failure of the body to maintain its normal core temperature when exposed to a high environmental temperature (i.e., “classic heat stroke”) or from strenuous exercise (i.e., “exertional heat stroke”) (1). There is no universally accepted definition of heat stroke. Based on the pathophysiology of heat stroke, Bouchama and Knochel (1) proposed to define heat stroke as “a form of hyperthermia associated with a systemic inflammatory response leading to a syndrome of multiorgan dysfunction in which encephalopathy predominates.” Heat stroke is often clinically defined as “a core body temperature that rises above 40°C and that is accompanied by hot, dry skin and central nervous system abnormalities such as delirium, convulsions, or coma” (1, 2). However, given the occurrence of multiple organ dysfunction in heat stroke victims, the Japanese Association for Acute Medicine (JAAM) recommended heatstroke clinical criteria to include “the presence of renal and hepatic complications and disseminated intravascular coagulation (DIC)” (3). The JAAM criteria do not include temperature “because elderly people often do not present with high temperature even when they are already suffering from heat stroke” (3). Heat stroke can lead to organ damage or death, and the hospital mortality rate in classic heat stroke ranges from 10% to 65%, whereas that of exertional heat stroke is 3% to 5% (4). An average of 702 heat-related deaths occurred in the United States annually during 2004–2018 (5) (we assume representing mostly cases of classic heat stroke). “Prompt recognition and effective body cooling will in most cases rapidly reverse heat-induced organ dysfunction. However, body cooling alone may not suffice to effect a full recovery, and prompt administration of adjuvant treatments may be critical for survival” (4). Several novel treatments are currently being explored for heat stroke, notably to address gut permeability and to reduce systemic inflammatory markers (4).

Myalgic encephalomyelitis/chronic fatigue syndrome (ME/CFS) is a debilitating multi-system disease of unclear etiology (6–10). The most common peri-onset events reported by patients are infection-related episodes, stressful incidents, and exposure to environmental toxins (11). “Impaired function, post-exertional malaise, and unrefreshing sleep” are considered to be the core symptoms (12, 13). Post-exertional malaise refers to an exacerbation of some or all of an individual's ME/CFS symptoms after physical or cognitive exertion, or orthostatic stress that leads to a reduction in functional ability. The severity of the symptoms varies, where “very severely affected patients experience profound weakness, almost constant pain, severe limitations to physical and mental activity, sensory hypersensitivity (light, touch, sound, smell, and certain foods), and hypersensitivity to medications” (14, 15). The illness can be completely incapacitating (16) and “at least one-quarter of ME/CFS patients are house- or bedbound at some point in their lives” (12). Patients with milder symptoms experience a significant reduction in their previous level of functioning (8). The illness progresses over time, shifting from an early hypermetabolic state to a hypometabolic state with low energy production (17). ME/CFS is often dismissed by physicians because standard urine and blood laboratory tests produce normal results, thereby multiplying patients' suffering (18). Deaths from ME/CFS are rare (19), however, the prevalence of the disease is estimated to be at least 1.5 million cases in the United States alone (20). There are currently no effective treatments for ME/CFS (21–25).

In the following sections, we provide an overview of key pathophysiological mechanisms in heat stroke, such as splanchnic vasoconstriction, increased gut permeability, gut-related endotoxemia, systemic inflammatory response, central nervous system dysfunction, blood coagulation disorder, endothelial-cell injury, and mitochondrial dysfunction - and relate these mechanisms to similar findings from ME/CFS research. In addition, we also describe the results of initial heat stress gene transcriptome studies and the parallel findings from ME/CFS studies. Finally, we summarize key predisposing factors for both illnesses.

The central thesis of this paper is that the lessons from the human studies and animal models of heat stroke can contribute to the understanding and treatment of ME/CFS. Although this thesis might seem unlikely given the important differences in the onset and clinical outcomes between these two conditions, it is derived from a broader hypothesis that supports the notion of common pathways in the physiological responses to very different forms of stressors and disease manifestations (26–30). The paper serves as an element of a wider enquiry into commonalities in the pathology of illnesses induced by physical, infectious, and/or emotional stressors including heat stroke, ME/CFS, prolonged critical illness (chronic intensive care unit [ICU]), post intensive care syndrome (PICS), cancer-related fatigue, post-viral fatigue, post-acute coronavirus disease 2019 [COVID-19] syndrome (PACS), and long-COVID-19. The paper is in line with a history of research comparing ME/CFS with other conditions (including fibromyalgia, multiple sclerosis, and rheumatoid arthritis) to derive lessons for understanding ME/CFS.

## Pathophysiological Mechanisms

**Figure 1:**
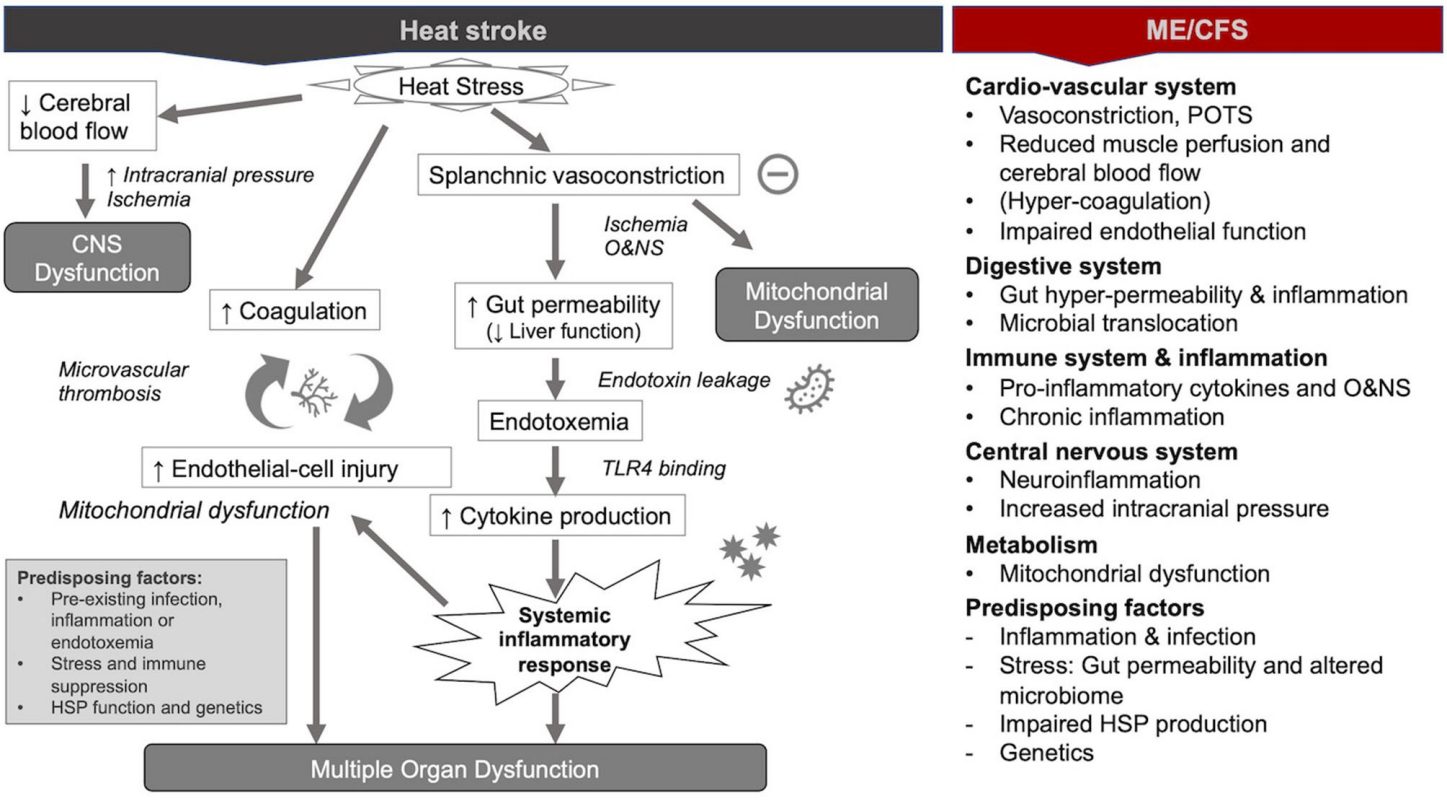
Pathophysiological mechanisms during heat stroke and similar mechanisms in myalgic encephalomyelitis/chronic fatigue syndrome ME/CFS [modified from (36)].

Since the 1990s, human and animal model studies have shown that endotoxemia of intestinal origin (i.e., the presence of endotoxins leaked from the intestines into circulation) may play an important role in the pathophysiological mechanisms of heat stroke (1, 2, 31–33). This evidence culminated in the *dual pathway model* of heat stroke, which proposes that heat stroke can be triggered by two sequential but independent pathways (31, 34): (i) the *heat toxicity pathway*, referring to cytotoxicity resulting directly from hyperthermia (e.g., protein denaturation, organ damage, and interruption of critical cellular processes because of heat), and (ii) the *endotoxemia pathway* (or heat sepsis pathway), involving splanchnic vasoconstriction, gut hyper-permeability (“leaky gut”), and endotoxemia culminating in systemic inflammation (or sepsis) (Figure 1). Whereas heat toxicity was traditionally considered the primary determinant of morbidity and mortality in heat stroke, it is now increasingly recognized that this pathway occurs only at temperatures above 42°C. Conversely, the endotoxemia pathway can provoke heat stroke at temperatures between 40 and 42°C (31, 33). In other words, the endotoxemia pathway precedes and acts largely independently of the heat toxicity pathway in the pathology of heat stroke (31). Moreover, the endotoxemia pathway is increasingly considered as the lead driver of severe organ damage and the main cause of death in patients suffering from heat stroke (35).

In the following paragraphs, we provide an overview of the mechanisms of the modern paradigm of heat stroke with a focus on the endotoxemia pathway. Moreover, we describe similar findings from ME/CFS. Our focus on the similarities between the two conditions does not intend to negate the disease-specific mechanisms. Instead, it intends to highlight possible lessons from heat stroke for understanding ME/CFS.

### Splanchnic Vasoconstriction and Hypoxia

#### In Heat Stroke

The primary response of the cardiovascular system to an increase in the ambient heat or heat from exertion is an increase in the blood flow to the skin (i.e., cutaneous vasodilation) to dissipate the excess heat. The modern paradigm of heat stroke suggests that the maintenance of a constant blood pressure implies/requires a reduction in blood flow to internal organs (i.e., splanchnic vasoconstriction) (1, 32, 36–38). However, splanchnic hypo-perfusion is not unique to heat stroke and common to critical illnesses (i.e., severe injury or infection) (39, 40), and may thus be a response to severe stress in general. In any case, the resulting hypoxia has important consequences on the gut and brain during heat stroke (as shown below) and promotes liver damage. In addition, the strain on the cardiovascular system often results in syncope and orthostatic intolerance in heat stroke (36).

#### In ME/CFS

Akin to heat stroke, several new hypotheses of ME/CFS suggest that vasoconstriction and hypoxia are central to its pathology—respectively focused on the consequence of ß2 adrenergic and M3 acetylcholine receptor autoantibodies (41, 42), a potential reduction of ACE2 expression (43), and an abnormal immune response with a concomitant impact on endothelial function (44). In addition, postural orthostatic tachycardia syndrome (POTS) is a common comorbidity of ME/CFS (45), which is a complex disorder of the vascular system also associated with gastrointestinal dysfunctions (46, 47). Impaired blood pressure variability and arrythmia have been observed in the patients with ME/CFS (48, 49).

**In summary**, vasoconstriction and hypoxia would appear to be common to both heat stroke in at least a subset of patients with ME/CFS—despite the difference in onset events of these two conditions. Research from critical illnesses (beyond heat stroke) suggests that splanchnic vasoconstriction is a general response to stressors. Vasoconstriction and hypoxia have far-reaching implications for organs and tissues.

### Gut Permeability and Endotoxemia

#### In Heat Stroke

Animal models of heat stroke have shown that ischemia resulting from reduced blood flow to the gut causes mucosal damage and a weakening of the tight junctions in the gut—likely *via* the action of oxidative and nitrosative stress (O&NS) (1, 4, 31–33, 35–38, 50, 51)—as well ATP depletion and local acidosis (50) induced by hypoxia. The result is a leaky gut characterized by a reduction of intestinal immunity and increased permeability of the intestines (1). This condition promotes gram-negative bacteria (i.e., lipopolysaccharides, LPS) that are normally contained in the gut to enter blood circulation (1, 4, 31–33, 35–38, 50, 52). Studies have shown that in the case of heat stroke, the liver is unable to effectively clear endotoxin (31), notably because of poor circulation in the venous system that returns blood from the digestive tract and spleen to the liver (37, 50). The resulting endotoxemia is assumed to trigger a systemic inflammatory response syndrome (SIRS) in heat stroke (as shown below) (4, 31–33, 35, 36, 53). Furthermore, findings from critical illness suggest that additional forms of intestinal injury play a role in driving critical illness, including the erosion of the mucus barrier, a shift in the composition and virulence of intestinal microbes, and the inability of the host epithelium to regulate its proliferative and apoptotic response (39, 54). These elements are thought to contribute to a vicious cycle of inflammation and intestinal injury. Moreover, the reduction in gut motility, the formation of toxic gut-derived lymph, and a decrease in the secretion of gastrointestinal hormones could contribute to multi-organ dysfunction in critical illness (55, 56).

Critically, normalization of the core temperature during heat stroke through cooling does not necessarily imply a reversion to normal gut permeability or the end of endotoxemia (1). Some researchers have thus suggested and tried to repair gut function to treat heat stroke (38). This includes the administration of nutritional supplements, such as glutamine and bovine colostrum with some promising results (33, 50, 57, 58). Additionally, the use of prebiotics and probiotics is being explored as an avenue for treatment (38, 50). Others have had success in limiting intestinal injury with the use of 4-phenylbutyrate to interrupt the endoplasmic reticulum stress-mediated apoptosis pathway of intestinal cells (59). Similarly, others have studied the use of xanthine oxidase inhibitor (allopurinol) to protect the integrity of tight cell-to-cell junctions in the gut of rat models (51). The inhibition of endotoxemia by pre-treatment with LPS-specific antibodies and corticosteroids to counteract LPS was shown to protect animals from lethal heat stress (31, 60). Readers are referred to the reviews for details on the results of human and animal studies (35, 38, 50, 61).

#### In ME/CFS

Echoing the endotoxemia model of heat stroke, ME/CFS researchers have hypothesized that the translocation of gut microbes or toxins from the gastrointestinal tract and the resulting inflammation is central to ME/CFS (62–66). Studies have found gut hyper-permeability (63) and gut inflammation (67) in ME/CFS as well as increased levels of the antibodies to LPS (IgM and IgA) in serum of patients with ME/CFS (the prevalence of patients with CFS with abnormally increased IgM and IgA levels: 40% and 66.7%) (68). Moreover, researchers have induced fatigue in mice through the administration of LPS (69). Finally, studies have shown that the normalization of leaky gut in ME/CFS by administration of natural anti-inflammatory and antioxidative substances is accompanied by a clinical improvement (70). Readers are referred to the reviews for a detailed discussion on the implication of gut permeability on the immune system and mitochondrial function in ME/CFS (7, 71).

It is worth noting that leaky gut is distinct from irritable bowel syndrome (IBS), although the former is believed to contribute to the latter (72). Leaky gut is related to changes in the permeability of the tight junctions in the gut barrier, which can be observed in the absence of symptoms (73). In research, changes in the gut barrier have been measured in urine using sugar probes of different molecular sizes, and in the circulation, using intestinal fatty acid binding protein (iFAB), and claudine-3 (74, 75). iFAB is an intracellular protein in the intestine wall and supports the transportation of fatty acid across the gut barrier. Claudine-3 is located between the tight junctions and functions as a “glue” to preserve the integrity of the gut barrier. Leaky gut is associated with diseases beyond the gut, such as diabetes (76) and autoimmune diseases (77, 78). Conversely, IBS is related to gut dysbiosis and is primarily associated with gastrointestinal discomfort (79). Whereas IBS is widely recognized as a clinical entity by the medical profession, leaky gut is largely considered the domain of alternative medicine (80).

**In summary**, gut hyper-permeability and endotoxemia would appear to be common to both heat stroke and at least a subset of patients with ME/CFS patients—despite the difference in onset events of these two conditions. These mechanisms have far-reaching implications on various physiological systems, including inflammation (as shown below). The results of human and animal experiments to repair gut function and mitigate endotoxemia in heat stroke might inform and provide new therapeutic avenues for ME/CFS.

### Systemic Inflammatory Response

#### In Heat Stroke

Bouchama and Knochel suggest that the inflammatory response during the initial “acute-phase of heat stress” is mediated primarily by interleukin-6 (IL-6) and other pro-inflammatory cytokines, which serve to protect against tissue injury and to promote repair. Indeed, IL-6 and other cytokines induced by exposure to heat stress appear to mediate fever, leukocytosis, increased synthesis of acute-phase proteins, muscle catabolism, stimulation of the hypothalamic–pituitary–adrenal axis, and the activation of leukocytes and endothelial cells (1). However, an exaggeration of the acute-phase response can precipitate a maladaptive progression from *heat stress* to *heat stroke*. Specifically, leaky gut (as described above) hastens local inflammation toward the “exaggerated” systemic inflammatory response that underpins heat stroke (1). The field of exercise immunology has demonstrated that LPS leaking from the gut activates toll-like receptor (TLR) 4 on immune and intestinal epithelial cell membranes. This activation results in the production of pro-inflammatory cytokines, e.g., IL-6, IL-1β, and tumor necrosis factor (TNF)-α (31, 33, 35, 50). Moreover, the animal models of heat stroke have shown that, when LPS is present, NLRP3 (NOD-, LRR-, and pyrin domain-containing protein 3) inflammasomes are activated and drive neuroinflammation (81). A vicious cycle centered around intestinal injury ensues in heat stroke involving the release of nitric oxide, the leakage of gut-related endotoxins, and increased production of inflammatory cytokines (e.g., TNF-α, IL-1β, and IFN-γ) (35). Anti-inflammatory cytokines (IL-10, IL-1 receptor antagonist (IL-1ra), and TNF receptors p55 and p75) are also elevated, but to a lesser degree (2). The mode of action of cytokines in a heat stroke remains to be fully understood (33, 36). However, the increase in inflammatory cytokine levels is associated with inflammation-associated injury and refractory immunosuppression (1, 36) and can induce fever when transported to the brain (1, 82). Mouse models indicate that glia cells are a likely source of cytokine and chemokine production (36, 83).

Critically, normalization of the core temperature does not necessarily inhibit the systemic inflammatory response in heat stroke (1). Researchers have thus suggested and tried the modulation of the inflammatory response as a form of heat stroke treatment and prophylaxis, including with the administration of IL-1 receptor antagonists (81, 84), corticosteroids (60, 85), activated protein C (86), and the inhibition of nuclear factor-kB activity (1). Others have used oleoylethanolamide to inhibit LPS-induced TLR-4 stimulation of the immune response (50, 87). Blood purification therapy (53) and bone marrow-derived mononuclear cell therapy (88) to reduce pro-inflammatory cytokines have been suggested or tested in clinical trials. Other clinical trials have tested the efficacy of antioxidants and anti-inflammatories, such as quercetin (89). One study found that parenteral administration of ascorbate attenuated heat stroke-induced systemic inflammation in mice (90). Readers are referred to the reviews for details on the results of human and animal heat stroke studies (35, 50).

#### In ME/CFS

Akin to heat stroke, researchers have found that chronic inflammation also underlies ME/CFS (91–95), including inflammation of the brain—hence the name myalgic encephalomyelitis (96, 97). Moreover, similarly to heat stroke, O&NS has been documented in ME/CFS (98) and the cytokine profile of patients with ME/CFS differs from healthy controls (99, 100). Researchers have also linked NLRP3 inflammasomes with inflammation in ME/CFS (101). Some researchers have theorized that the complex pathways between these elements (O&NS, cytokines, and inflammation) underpin the metabolic dysfunction characteristic of the disease (102), including by hindering mitochondrial (103) and hypothalamic function (104, 105). Significantly, researchers suggest “the possibility of a vicious cycle of immune-dysregulation and gut dysbiosis accompanied by poor physiological gut function” perpetuating a disease state in ME/CFS (7)—as is the case for heat stroke. Readers are referred to the numerous studies and reviews for a detailed discussion on the relationship between redox imbalance, cytokines, and inflammatory pathways in ME/CFS (7, 101, 106, 107).

**In summary**, inflammation associated with cytokines and O&NS is central to both heat stroke and ME/CFS—despite the difference in onset events of these two conditions. Moreover, a vicious inflammatory cycle involving altered gut-blood barrier function (leaky gut) may be a central element of both heat stroke and some patients with ME/CFS. The results of human and animal experiments to modulate the inflammatory response in heat stroke might inform and provide new therapeutic avenues for ME/CFS.

### Central Nervous System Dysfunction

#### In Heat Stroke

Delirium, convulsions, and coma are common symptoms in the clinical presentation of heat stroke. The mechanisms of central nervous system injury in heat stroke are not well understood but are likely due to the combination of ischemia, hypoxia, thermolysis (Tc > 42°C), and hemorrhage (108–110). The animal models of heat stroke identified ischemia resulting from decreased cerebral flow and increased intracranial pressure as causing extensive cerebral neuronal injury (36). Moreover, a decrease in brain blood supply and an increase in intracranial pressure are followed by a compromised blood-brain barrier and an increase in circulatory pyrogens (37). In other words, analogous to the consequences of hypo-perfusion in the gut, reduced blood flow to the brain can lead to hypoxia, inflammation, and hyper-permeability within the brain, facilitating protein and pathogen leakage from the systemic circulation into the brain (32, 36, 37, 111). The severity of this mechanism in heat stroke can lead to brain injury.

#### In ME/CFS

Central nervous system dysfunctions are present in ME/CFS, albeit in a different form. Brain fog and hypersensitivity to noise and light are common symptoms in patients with ME/CFS (8, 13). However, the mechanisms in ME/CFS are arguably even less well understood than in heat stroke. Brain imaging studies revealed significant neuroinflammation (112–114) and white matter abnormalities (115), which are confirmed by autopsies (116). As mentioned above, a new unifying hypothesis of ME/CFS suggests that vasoconstriction and hypoxia in muscle and brain underpin ME/CFS, including cognitive impairments (41, 42). Studies have shown reduced cerebral blood flow during tilt tests (117). Akin to heat stroke, some patients with ME/CFS present signs of increased intracranial pressure (118, 119), consistent with altered blood flow. Alternatively, others have hypothesized that ME/CFS is promoted by gut microbes or toxin translocation from the gastrointestinal tract into other tissues, including the brain (62). The central nervous system is generally an immune-privileged site (i.e., protected from the peripheral immune system) (120), but pathogens that reach the brain can cause devastating inflammation (121).

**In summary**, central nervous system dysfunctions are central to both heat stroke and ME/CFS, albeit in different forms and to different degrees. Neuroinflammation, decreased blood flow to the brain and increased intracranial pressure might be common to both heat stroke and some ME/CFS patients. The animal models of heat stroke may inform the understanding of central nervous system dysfunction in ME/CFS.

### Coagulation Disorders and Endothelial-Cell Injury

#### In Heat Stroke

Both the endotoxemia and the heat toxicity pathways are believed to contribute to coagulopathy dysfunction and endothelial-cell injury in heat stroke. First, intestine-derived endotoxins and cytokines activate vascular endothelial cells and coagulation factors to amplify the coagulation cascade (32, 35), which triggers vascular endothelial cell injury (122, 123). Moreover, the increase in core temperature directly leads to vascular endothelium damage (2), which in turn drives coagulation (32, 36). Bouchama and Knochel (1) describe a complex interplay among the cytotoxic effect of the heat and the inflammatory and coagulation responses of the host. Together these mechanisms result in a positive feedback loop, causing occlusion of the arterioles and capillaries (i.e., microvascular thrombosis) (32, 53, 90, 122). The vascular endothelial cell injury in heat stroke has been substantiated by evidence of differential expression of exosome lncRNAs and miRNAs (122).

Critically, normalization of the core temperature does not necessarily deactivate the coagulation process (1). Researchers have thus suggested anticoagulation therapy (53) with some initial positive results (124). Others have found that dexmedetomidine protects endothelial glycocalyx and increases the survival of rats during heat stroke (125). Readers are referred to the reviews for details on the results of human and animal studies (35, 38, 50).

#### In ME/CFS

Akin to heat stroke, hyper-coagulation was also suggested to be a contributing factor in ME/CFS (126–128), but the findings have not been consistent across the studies (129). The lessor importance or absence of coagulation dysfunctions in ME/CFS compared with heat stroke may be explained by the fact that the “heat toxicity pathway,” one of the drivers of the “positive feedback loop” driving coagulopathy dysfunction in heat stroke, is absent in ME/CFS.

Studies have shown that endothelial function is impaired in ME/CFS, both in large vessels and in microcirculation (130, 131). As in heat stroke, these findings were substantiated by indirect evidence related to the expression of miRNAs relevant to endothelial function (132). A new hypothesis for ME/CFS suggests that endothelial senescence underpins ME/CFS by disrupting the intestinal and blood-brain barriers (62).

**In summary**, coagulopathy dysfunction and endothelial-cell injury may be common to both heat stroke and at least a subset of patients with ME/CFS, despite the difference in onset events of these two conditions. The results of human and animal experiments to address coagulation disorders and endothelial-cell injury in heat stroke might inform and provide new therapeutic avenues for ME/CFS.

### Mitochondrial Dysfunction

#### In Heat Stroke

There are only few studies that assess mitochondrial function in heat stroke. ATP depletion (36) as well mitochondrial swelling and vacuolization in the epithelial cells of the gut have been observed in the mice models of heat stroke (52). Moreover, animal studies have shown that apoptosis of vascular endothelial cells in heat stroke was the deleterious consequence of reactive oxygen species (ROS)-induced p53 translocation into mitochondria (133). Finally, it was shown *in vitro* that higher temperatures can directly affect the viscosity of mitochondria (134).

#### In ME/CFS

Akin to heat stroke, the immuno-inflammatory pathways associated with O&NS are thought to be one of the possible mechanisms driving mitochondrial dysfunction in ME/CFS (103, 107). [Alternatively, mitochondrial dysfunction could be caused by viral reactivation in ME/CFS (135, 136)]. Impaired energy production (97, 137) and reduced mitochondrial activity (138–140) are central to the pathology of ME/CFS (7). Researchers have observed mitochondrial fragmentation (135) and impaired pyruvate dehydrogenase function as well as lactate degradation deficiencies in ME/CFS (141). Moreover, irregularities in the metabolites of patients with ME/CFS (141, 142) suggest that patients experience a hypometabolic or “dauer” state (143) maintained by mitochondria; an evolutionary adaptation to protect the cells and hosts from harm (c.f., “cell danger response”) (30, 144, 145). Readers are referred to the reviews for a detailed discussion on the relationship between the immune system and mitochondrial function in ME/CFS (7, 71).

**In summary**, mitochondrial dysfunction may be common to both heat stroke and ME/CFS, despite the difference in onset events of these two conditions. Mitochondrial dysfunction can be driven by immuno-inflammatory and O&NS pathways. The animal models of heat stroke may inform the understanding of mitochondrial dysfunction in some cases of ME/CFS.

### Altered Gene Transcription

#### In Heat Stroke

A human transcriptome study performed by Bouchama et al. using peripheral blood mononuclear cells (PBMCs) of healthy volunteers exposed to short but extreme environmental heat treatment found “downregulation of most of the genes involved in the respiratory chain […] with a predicted decrease in ATP production and increase in oxidative stress.” The findings suggested that the “shift to glycolysis as a source of energy” and “inhibition of mitochondrial respiration” were sustained at least an hour after the end of the heat treatment. In addition, there was a “downregulation of ubiquitin conjugation and proteasome genes with a predicted decrease of protein refolding, mono- and poly-ubiquitination, and antigen presentation” as well as “decreased expression of the major histocompatibility I and II complexes genes, suggesting reduced antigen presentation” that persisted at least an hour after the end of treatment. To be clear, the volunteers did not experience *heat stroke* (only *heat stress*), but nonetheless, the study showed an adaptive response of mitochondria to heat stress - namely, altered protein synthesis, and reduced expression of genes related to immune function - to persist at least an hour after the heat stress ended. The authors could not explain why cellular stress response to heat would involve a “switch to a less efficient form of metabolism”(146).

#### In ME/CFS

Transcriptome studies have generally found altered gene expression in patients with ME/CFS (147–154). Researchers, for example, identified changes in the expression of genes in peripheral immune cells related to inflammation and mitochondrial function (153) as well as to oxidative stress (149) and immune function (149, 150, 154). Others have found specific microRNA expression patterns associated with specific symptom severities (155). Readers are referred to the reviews for a detailed discussion on alterations in gene expression in ME/CFS (95).

**In summary**, initial gene transcriptome studies would appear to suggest overlaps in alterations of gene expressions between the two conditions. Specifically, alterations in genes related to mitochondrial and immune function may be common to both heat stroke and some patients with ME/CFS. Future transcriptome studies of heat stroke may inform the understanding of ME/CFS.

### Predisposing Factors

#### In Heat Stroke

Young children, the elderly, and patients with chronic diseases are most susceptible to classic heat stroke. Further intrinsic risk factors for classic heat stroke include underlying illness, cardiovascular insufficiency, concurrent infections, and an immune-compromised state (36).

However, the predisposing factors for exertional heat stroke—which affects young and apparently healthy and active individuals—are less clear. Similar to classic heat stroke, researchers have suggested that prior bouts of illness, infection or inflammation, preexisting barrier dysfunction, and a compromised immune system may increase the risk of exertional heat stroke (50, 156, 157). Specifically, prolonged stress exposure and/or suboptimal dietary intake - which suppress the immune system and alter the microbiome of the host, thus depleting its immune and epithelial barrier functions - are thought to be the determining factors in the susceptibility to exertional heat stroke (31, 50). Accordingly, some researchers have suggested that stress and lifestyle factors set the stage for a single event that promotes LPS translocation (such as exertion) to overwhelm the suppressed immune system (31). In the central circulation, LPS can be bound to LPS-specific antibodies, monocytes, or high-density lipoprotein and transported to the liver for removal by the reticuloendothelial system (31, 34). Immune suppression compromises LPS removal by monocytes and LPS-specific antibodies, leading to an increase in the concentration of LPS in the circulation (34, 158) and thus susceptibility to endotoxemia.

In terms of prior infection, a hypothesis derived from exercise immunology research suggests that the immune system is suppressed temporarily immediately post-intense exercise, which provides a window for dormant viruses to be reactivated to cause an infection (c.f. “open window theory”) (159). More specifically, a group of researchers has shown that the increased incidence of illness in response to excessive exercise is not due to immunosuppression *per se*, but rather to an altered focus of the immune function: an upregulation of humoral immunity and suppression of cell-mediated immunity (160).

Regarding gender bias, a review of 22 studies found that the overall risk for heat illnesses was 2.64 times higher in men than in women, but the authors suggest this could relate to sex-associated behavioral differences rather than physiology (161). Indeed, women are disadvantaged in terms of thermoregulation because of the smaller body surface area (BSA) to mass ratio, a higher percentage of body fat, and lower maximal oxygen consumption (VO_2_max) (158). A study showed that greater heat intolerance in women relative to men is largely explained by VO_2_max (162). A review of 24 studies of gender differences in the armed forces suggest that being a female was associated with a greater risk of exertional heat illnesses, but men experienced a slightly higher incidence of exertional heat stroke (163)—perhaps also because of behavioral differences.

Failure to increase the expression of heat shock proteins (HSPs) has been implicated in the pathogenesis of heat stroke (146). HSPs play a central role in the maintenance of endothelial and epithelial barrier function (164, 165). Conditions associated with a low level of expression of HSPs, for instance, aging, lack of acclimatization to heat, and certain genetic polymorphisms, may favor the progression from heat stress to heat stroke (1). Initial studies have shown that selective pharmacologic induction of the expression of HSPs (e.g., with alpha-lipoic acid) can protect cells from apoptosis and inflammatory responses induced by heat stroke (166).

Finally, Bouchama and Knochel suggest that “genetic factors may determine the susceptibility to heat stroke; candidate susceptibility genes include those that encode cytokines, coagulation proteins, and heat-shock proteins involved in the adaptation to heat stress” (1). Specifically, TLR4 polymorphisms may be a genetic factor predisposing to mortality during the systemic inflammatory response to heat stroke (36). However, the importance of genetic factors as a risk for heat stroke remains unresolved.

#### In ME/CFS

Akin to exertional heat stroke, the predisposing factors for ME/CFS are not clear. Certainly, existing inflammation or infection is likely a risk factor (167). Echoing the “open window theory” of exercise immunology, it has long been suggested that ME/CFS results from bacterial or viral reactivation in the context of a suppressed immune system (168, 169), particularly the reactivation of Epstein-Barr virus (EBV) and cytomegalovirus (CMV) (170, 171). Cumulative life stress is considered a predisposing factor for ME/CFS (172), which is also thought to alter gut permeability and thus immune function (173). As in heat stroke, the microbiome disturbances and dysbiosis are associated with ME/CFS pathology (147, 174–179). Gender is a risk factor for ME/CFS: the female-to-male ratio ranges from 2:1 to 6:1 (12).

Moreover, the studies have shown that ME/CFS is characterized by impaired HSPs production (180) which, combined with O&NS and low-grade inflammation, could explain muscle dysfunction and exercise intolerance (181, 182). Akin to heat stroke, it has been suggested (but not yet trialed) to incorporate the upregulation of HSP into future treatments for ME/CFS (183).

Finally, research suggests there may be a genetic element in the pathogenesis of ME/CFS, such as polymorphism in genes related to immunomodulatory response (184, 185), hormone action (184, 186), and metabolic kynurenic pathways (187). However, genetic associations are not consistent (188) and studies have had quality-control issues (188, 189).

**In summary**, there are important similarities in predisposing factors for heat stroke and ME/CFS. Pre-existing inflammation or infection may make individuals susceptible to the conditions. Viral reactivation may also play a role in both conditions. Both are associated with immune suppression. Parallel hypotheses suggest that cumulative stress and associated degradation of microbiome health could set the stage for increased susceptibility for both conditions. There is a female predominance in ME/CFS; physiological differences may make women intrinsically more susceptible to exertional heat illnesses than men. In addition, insufficient expression of HSPs has been identified as a factor in both conditions. Finally, genetics are thought to be a risk factor for both conditions, but studies are inconclusive.

## Discussion

We suggest that the research on heat stroke might provide important lessons for understanding and treating ME/CFS. First, the findings from heat stroke confirm the possibility of systemic inflammation that is not associated with an exogenous pathogen (but rather with endogenous toxins). Heat stroke is primarily an inflammatory response following an external stressor (i.e., an increase in ambient temperature or over-exertion during exercise). This insight is likely important for the broader acceptance of ME/CFS as a disease that does not necessarily result from an exogenous pathogen.

Second, the findings from heat stroke describe a sequence and causality between physiological disturbances in various body systems, including the cardiovascular, gastro-intestinal, immune, and central nervous systems. The sequential pathway from splanchnic vasoconstriction to hypoxia, redox imbalance, and gut hyper-permeability (or intestinal injury) has been consistently documented in both classic and exertional heat stroke. The resulting endotoxemia, cytokine production, systemic inflammation, and decreased cerebral flow are now understood to underlie mitochondrial, central nervous system, and multi-organ dysfunction in heat stroke. This knowledge of the sequence and causality across systems is likely relevant for understanding ME/CFS because of the overlaps in anomalies across the two illnesses.

Third, heat stroke provides a model for disease perpetuation. The heat stroke literature describes a “vicious cycle” centered around intestinal injury involving the release of nitric oxide, the leakage of endotoxins, and the increased production of inflammatory cytokines. This model may also be relevant for understanding the perpetuation of disease state in ME/CFS, as these elements have also been documented in ME/CFS. However, the prevalence of endotoxemia in ME/CFS requires further study to estimate for what share of patients with ME/CFS a similar “vicious cycle” might apply.

Fourth, heat stroke research—combined with the findings from critical illness in general—suggests that intestinal injury resulting from splanchnic hypo-perfusion is a common stress response. This insight could help to understand how a variety of triggers [i.e., physical, infectious, and/or emotional stressor(s)] can result in homeostatic imbalance by initiating intestinal injury. Incidentally, intestinal permeability and microbial translocation are hypothesized to contribute to the pathology in multiple sclerosis (190), rheumatoid arthritis (191), fibromyalgia (192), and Hashimoto's thyroiditis (193)—diseases for which previous studies have described shared pathophysiological mechanisms with ME/CFS (194–198). The endotoxemia and inflammatory pathways described in these diseases echo the patterns observed in heat stroke. A comparative assessment of the overlaps (and differences) between these illnesses would surely provide an important contribution toward resolving these diseases.

Fifth, the results of therapeutic studies for heat stroke in animals and people may indicate promising avenues for the treatment of ME/CFS. Heat stroke researchers have tested the use of pharmacological agents and supplements to repair the immune and barrier function of the gut, mitigate endotoxemia, and modulate the inflammatory response. By building on these findings, researchers may identify therapeutic avenues with potential for ME/CFS. The treatment of heat stroke is incomplete and remains an active area of research; a collaboration between ME/CFS and heat stroke researchers would likely benefit both fields.

The findings from heat stroke may provide lessons for understanding both the early and late stages of ME/CFS. Much of heat stroke literature describes the mechanisms occurring within the first 72 h of illness following heat stress. Specifically, adaptive mechanisms intended to deal with stressors are “exaggerated” in certain individuals, thereby overwhelming the homeostasis of the body. Similar exaggerated adaptative responses to external stressors and overwhelmed homeostasis may explain the early stage of ME/CFS. Conversely, the detailed findings from heat stroke regarding the mechanisms that prevent a reversion to a normal homeostatic state even once the stressor is removed (c.f. endotoxemia, O&NS, and pro-inflammatory cytokines) may help to understand the late stages of ME/CFS in at least a subset of patients with ME/CFS.

The existence of overlaps between heat stroke and ME/CFS might initially appear unlikely because heat stroke is a critical illness requiring intensive care, whereas ME/CFS is a chronic illness that debilitates patients for months and years. Moreover, the lack of a disease-specific biomarker and heterogeneity in the clinical symptoms of patients with ME/CFS (resulting in misclassification/misdiagnosis) - combined with the small sample size of many existing studies, pose a challenge in the assessment of overlaps between the two conditions. Yet our suggestion of overlaps between ME/CFS and heat stroke is in line with earlier hypotheses of common pathways in the physiological responses to very different forms of stressors (26–30). A difference in the severity of the vasoconstriction and “endotoxemia pathway” (i.e., intestinal injury) between the two illnesses might explain the difference in the critical/chronic natures of the illnesses. Whereas in heat stroke the vasoconstriction and “endotoxemia pathway” are so severe that they lead to brain injury and “heat sepsis,” the same mechanisms may be less severe but underpin brain fog and chronic inflammation in some patients with ME/CFS. Speculatively, at least a subgroup of patients with ME/CFS might be thought of experiencing a series of mild exertional heat strokes or a milder but chronic form of the pathological mechanisms witnessed in exertional heat stroke. Accordingly, post-exertional malaise - a key symptom of ME/CFS according to Canadian Consensus Criteria - could be the manifestation of accentuation of these mechanisms following exertion. Additional research is required to assess the relative severity of the pathophysiological mechanisms described above within heat stroke and ME/CFS, and identify a subgroup of patients where this hypothesis and the above lessons may be most applicable.

Heat stroke is not known to provoke ME/CFS. However, the patients surviving life threatening illnesses can develop PICS, i.e., “the cognitive, psychiatric, and/or physical disability after treatment in ICUs,” including ICU-acquired weakness (199, 200). PICS resembles ME/CFS and remains unresolved. It has been suggested that the persistence of endocrine disturbances observed during critical illness could also explain PICS (201). The similarities and differences between PICS and ME/CFS require further study. Auto-immunity—an important factor in ME/CFS pathology (91, 93, 197)—remains largely unexplored in heat stroke. However, auto-immunity has been associated with inflammatory cytokines and leaky gut (77, 78), which are present in heat stroke.

Neuroendocrine functions during heat stroke remain largely unexplored (31). However, it would seem plausible that the biphasic neuroendocrine pattern found in other critical illnesses might also exist in heat stroke. Specifically, an *acute* phase (in the first few hours or days) characterized by *increased* release of pituitary hormones, followed in some critical care patients by a *prolonged* phase (after a few days) characterized by the *suppression* of the release of pituitary hormones (202, 203). We have previously described the relevance of the mechanisms during the *prolonged* phase for ME/CFS (28)—notably the suppression of the pituitary gland's pulsatile secretion of tropic hormones (204), and a “vicious circle” between inflammation, O&NS, and low thyroid hormone function (205, 206). A compilation of the relevant findings from heat stroke described above (i.e., on the vascular, gastro-intestinal, immune, and nervous systems) and the relevant findings from critical illnesses described previously (i.e., neuroendocrine dysfunctions) might provide important insights into the mechanisms that prevent recovery in ME/CFS (28).

Finally, the lessons-learned from heat stroke for ME/CFS may also be relevant for the aftermath of the COVID-19 pandemic. Many patients with COVID-19 continue to experience a variety of debilitating symptoms despite successfully clearing the virus—termed “post COVID-19 syndrome” or “long COVID-19” —that resemble ME/CFS (207–215). Moreover, the patients with ME/CFS and COVID-19 recovery patients share “molecular signatures” (216), which suggest overlaps in immune and metabolic dysregulation (101).

## Conclusion

There may be overlaps in the pathophysiological mechanisms in heat stroke and ME/CFS, including splanchnic vasoconstriction, increased gut permeability, gut-related endotoxemia, systemic inflammatory response, central nervous system dysfunction, blood coagulation disorder, endothelial-cell injury, and mitochondrial dysfunction. Moreover, the pathophysiological mechanisms involved may be self-perpetuating in both conditions. Indeed, normalizing body temperature does not necessarily reverse the multiorgan dysfunction in heat stroke; similarly, ME/CFS persists even after the onset event (i.e., physical, infectious, and/or emotional stressor) has ended. A vicious cycle centered around intestinal injury involving the release of nitric oxide, the leakage of endotoxins, and increased production of inflammatory cytokines are central to heat stroke and have also been observed in some patients with ME/CFS. Moreover, initial gene transcriptome studies show that the alterations in gene expression related to mitochondrial and immune function may be common to both conditions. Finally, predisposing factors for heat stroke and ME/CFS appear to partly overlap, including prior inflammation and the altered gut microbiome. Genetics may contribute to the predisposition of individuals in both illnesses, but the existing evidence is inconclusive. Neuroendocrine functions during heat stroke remain largely unexplored. The lack of a disease-specific biomarker and heterogeneity in the clinical symptoms of patients with ME/CFS—combined with the small sample size of many existing studies—pose a challenge in the assessment of overlaps between these illnesses.

A full description of therapeutic trials for heat stroke and their relevance for ME/CFS is beyond the scope of the paper. However, therapeutic approaches to repair the immune and barrier function of the gut, mitigate endotoxemia, modulate the inflammatory response, and induce the expression of HSPs have been suggested or trialed for both conditions.

We suggest that lessons from the human studies and animal models of heat stroke might contribute to the understanding of ME/CFS. Specifically, the human studies and animal models of heat stroke confirm the possibility of systemic inflammation that is not associated with an exogenous pathogen. Moreover, they describe a sequence and causality between physiological disturbances in various body systems and provide an explanation for the self-perpetuation of the disease state that might also inform the understanding of ME/CFS. These studies—combined with the studies from critical illness in general—could help to understand how a variety of triggers (i.e., physical, infectious, and/or emotional stressor) might lead to homeostatic imbalance by initiating intestinal hyper-permeability (or intestinal injury). Moreover, given the similarities described above, an exhaustive analysis of the treatments already tried for heat stroke might help identify which ones could be redeployed to treat ME/CFS. Additional research is required to identify a subgroup of patients with ME/CFS where these lessons may be most applicable.

The existence of possible overlapping pathophysiological mechanisms between heat stroke and ME/CFS could further provide support for a broader hypothesis that the maladaptive mechanisms that prevent recovery in ME/CFS may be common to conditions induced by physical, infectious, and/or emotional stressors, such as heat stroke, prolonged critical illness (chronic ICU), PICS, cancer-related fatigue, post-viral fatigue, PACS, and long-COVID-19. We thus suggest that collaborative efforts should be sought among the researcher community across these conditions to better understand these conditions and identify treatments mitigating the functional disability that they induce.

## Data Availability Statement

The original contributions presented in the study are included in the article/supplementary material, further inquiries can be directed to the corresponding author/s.

## Author Contributions

DS wrote the first draft of the manuscript. All authors contributed to manuscript revision, read, and approved the submitted version.

## Funding

Open Medicine Foundation (JB) is acknowledged for support. NS acknowledges funding from the Polish National Agency for Academic Exchange (Reference Grant: PPN/ULM/2020/1/00069/U/00001) and FCT—Fundação para a Ciência e Tecnologia, Portugal (Ref. Grant: UIDB/00006/2020).

## Conflict of Interest

The authors declare that the research was conducted in the absence of any commercial or financial relationships that could be construed as a potential conflict of interest.

## Publisher's Note

All claims expressed in this article are solely those of the authors and do not necessarily represent those of their affiliated organizations, or those of the publisher, the editors and the reviewers. Any product that may be evaluated in this article, or claim that may be made by its manufacturer, is not guaranteed or endorsed by the publisher.

## Abbreviations

CMV: Cytomegalovirus
DIC: disseminated intravascular coagulation
EBV: epstein-barr virus
HSPs: heat shock proteins
IBS: irritable bowel syndrome
ICU: intensive care unit
IL: interleukin
JAAM: Japanese association for acute medicine
LPS: lipopolysaccharides
ME/CFS: myalgic encephalomyelitis/chronic fatigue syndrome
O&NS: oxidative and nitrosative stress
PACS: Post-acute COVID-19 syndrome
PICS: Post-intensive care syndrome
POTS: postural orthostatic tachycardia syndrome
ROS: reactive oxygen species
SIRS: systemic inflammatory response syndrome
TNF: tumor necrosis factor
TLR: toll-like receptor.

## References

[1] Bouchama A Knochel JP. Heat stroke. N Engl J Med. (2002) 346:1978–88. 10.1056/NEJMra011089 12075060

[2] Lugo-Amador NM Rothenhaus T Moyer P. Heat-related illness. Emerg Med Clin North Am. (2004) 22:315–27, viii. 10.1016/j.emc.2004.01.004 15163570

[3] Kondo Y Hifumi T Shimazaki J Oda Y Shiraishi SI Hayashida K. Comparison between the bouchama and Japanese association for acute medicine heatstroke criteria with regard to the diagnosis and prediction of mortality of heatstroke patients: a multicenter observational study. Int J Environ Res Public Health. (2019) 16:18. 10.3390/ijerph16183433 31527479 PMC6765926

[4] Epstein Y Yanovich R. Heatstroke. N Engl J Med. (2019) 380:2449–59. 10.1056/NEJMra1810762 31216400

[5] Vaidyanathan A Malilay J Schramm P Saha S. Heat-related deaths–United States, 2004–2018. MMWR Morb Mortal Wkly Rep. (2020) 69:729–34. 10.15585/mmwr.mm6924a1 32555133 PMC7302478

[6] Komaroff AL. Advances in understanding the pathophysiology of chronic fatigue syndrome. JAMA. (2019) 322:499–500. 10.1001/jama.2019.8312 31276153

[7] Missailidis D Annesley SJ Fisher PR. Pathological mechanisms underlying myalgic encephalomyelitis/chronic fatigue syndrome. Diagnostics. (2019) 9:3. 10.3390/diagnostics9030080 31330791 PMC6787592

[8] Nacul L Authier FJ Scheibenbogen C Lorusso L Helland IB Martin JA. European network on myalgic encephalomyelitis/chronic fatigue syndrome (EUROMENE): expert consensus on the diagnosis, service provision, and care of people with ME/CFS in Europe. Medicina. (2021) 57:510. 10.3390/medicina57050510 34069603 PMC8161074

[9] Cortes Rivera M Mastronardi C Silva-Aldana CT Arcos-Burgos M Lidbury BA. Myalgic encephalomyelitis/chronic fatigue syndrome: a comprehensive review. Diagnostics. (2019) 9:3. 10.3390/diagnostics9030091 31394725 PMC6787585

[10] Domingues TD Grabowska AD Lee JS Ameijeiras-Alonso J Westermeier F Scheibenbogen C. Herpesviruses serology distinguishes different subgroups of patients from the United Kingdom myalgic encephalomyelitis/chronic fatigue syndrome biobank. Front Med. (2021) 8:686736. 10.3389/fmed.2021.686736 34291062 PMC8287507

[11] Chu L Valencia IJ Garvert DW Montoya JG. Onset patterns and course of myalgic encephalomyelitis/chronic fatigue syndrome. Front Pediatr. (2019) 7:12. 10.3389/fped.2019.00012 30805319 PMC6370741

[12] Institute of Medicine. Beyond Myalgic Encephalomyelitis/Chronic Fatigue Syndrome: Redefining an Illness. Washington, DC: The National Academies Press (2015). 25695122

[13] Open Medicine Foundation. Symptoms of ME/CFS. (2020). Available online at: https://www.omf.ngo/symptoms-mecfs (accessed March 27, 2021).

[14] Centers for Disease Control Prevention. Clinical Care of Patients with ME/CFS–Severely Affected Patients. (2019). Available online at: https://www.cdc.gov/me-cfs/healthcare-providers/clinical-care-patients-mecfs/severely-affected-patients.html [accessed March 27, 2021].

[15] Williams LR Isaacson-Barash C. Three cases of severe me/cfs in adults. Healthcare. (2021) 9:2. 10.3390/healthcare9020215 33669438 PMC7920463

[16] Dafoe W. Extremely severe ME/CFS—a personal account. Healthcare. (2021) 9:504. 10.3390/healthcare9050504 33925566 PMC8145314

[17] Nacul L O'Boyle S Palla L Nacul FE Mudie K Kingdon CC. How myalgic encephalomyelitis/chronic fatigue syndrome (ME/CFS) progresses: the natural history of ME/CFS. Front Neurol. (2020) 11:826. 10.3389/fneur.2020.00826 32849252 PMC7431524

[18] Komaroff AL. Myalgic encephalomyelitis/chronic fatigue syndrome: when suffering is multiplied. Healthcare. (2021) 9:919. 10.3390/healthcare9070919 34356297 PMC8307552

[19] McManimen SL Devendorf AR Brown AA Moore BC Moore JH Jason LA. Mortality in patients with myalgic encephalomyelitis and chronic fatigue syndrome. Fatigue. (2016) 4:195–207. 10.1080/21641846.2016.1236588 28070451 PMC5218818

[20] Jason LA Mirin AA. Updating the national academy of medicine ME/CFS prevalence and economic impact figures to account for population growth and inflation. Fatigue: Biomed Health Behav. (2021) 21:1–5. 10.1080/21641846.2021.1878716

[21] Toogood PL Clauw DJ Phadke S Hoffman D. Myalgic encephalomyelitis/chronic fatigue syndrome (ME/CFS): where will the drugs come from? Pharmacol Res. (2021) :105465. 10.1016/j.phrs.2021.105465 33529750

[22] Richman S Morris MC Broderick G Craddock TJA Klimas NG Fletcher MA. Pharmaceutical interventions in chronic fatigue syndrome: a literature-based commentary. Clinic Therapeut. (2019) 41:798–805. 10.1016/j.clinthera.2019.02.011 30871727 PMC6543846

[23] Kim DY Lee JS Park SY Kim SJ Son CG. Systematic review of randomized controlled trials for chronic fatigue syndrome/myalgic encephalomyelitis (CFS/ME). J Transl Med. (2020) 18:7. 10.1186/s12967-019-02196-9 31906979 PMC6943902

[24] Castro-Marrero J Sáez-Francàs N Santillo D Alegre J. Treatment and management of chronic fatigue syndrome/myalgic encephalomyelitis: all roads lead to Rome. Br J Pharmacol. (2017) 174:345–69. 10.1111/bph.13702 28052319 PMC5301046

[25] Rowe PC Underhill RA Friedman KJ Gurwitt A Medow MS Schwartz MS. Myalgic encephalomyelitis/chronic fatigue syndrome diagnosis and management in young people: a primer. Front Pediatric. (2017) 5:121. 10.3389/fped.2017.00121 28674681 PMC5474682

[26] Arnett SV Clark IA. Inflammatory fatigue and sickness behaviour–lessons for the diagnosis and management of chronic fatigue syndrome. J Affect Disord. (2012) 141:130–42. 10.1016/j.jad.2012.04.004 22578888

[27] Craddock TJ Fritsch P Rice MA Jr del Rosario RM Miller DB. A role for homeostatic drive in the perpetuation of complex chronic illness: Gulf War Illness and chronic fatigue syndrome. PLoS ONE. (2014) 9:e84839. 10.1371/journal.pone.0084839 24416298 PMC3885655

[28] Stanculescu D Larsson L Bergquist J. Hypothesis: mechanisms that prevent recovery in prolonged ICU patients also underlie myalgic encephalomyelitis/chronic fatigue syndrome (ME/CFS). Front Med. (2021) 8:41. 10.3389/fmed.2021.628029 33585528 PMC7876311

[29] Stanculescu D Larsson L Bergquist J. Theory: treatments for prolonged ICU patients may provide new therapeutic avenues for myalgic encephalomyelitis/chronic fatigue syndrome (ME/CFS). Front Med. (2021) 8:556. 10.3389/fmed.2021.672370 34026797 PMC8137963

[30] Naviaux RK. Metabolic features of the cell danger response. Mitochondrion. (2014) 16:7–17. 10.1016/j.mito.2013.08.006 23981537

[31] Lim CL. Heat sepsis precedes heat toxicity in the pathophysiology of heat stroke-a new paradigm on an ancient disease. Antioxidants. (2018) 7:11. 10.3390/antiox7110149 30366410 PMC6262330

[32] Leon LR Helwig BG. Heat stroke: role of the systemic inflammatory response. J Appl Physiol. (2010) 10:1980–8. 10.1152/japplphysiol.00301.2010 20522730

[33] Ogden HB Child RB Fallowfield JL Delves SK Westwood CS Layden JD. The gastrointestinal exertional heat stroke paradigm: pathophysiology, assessment, severity, aetiology and nutritional countermeasures. Nutrients. (2020) 12:2. 10.3390/nu12020537 32093001 PMC7071449

[34] Lim CL Mackinnon LT. The roles of exercise-induced immune system disturbances in the pathology of heat stroke: the dual pathway model of heat stroke. Sports Med. (2006) 36:39–64. 10.2165/00007256-200636010-00004 16445310

[35] Ye N Yu T Guo H Li J. Intestinal injury in heat stroke. J Emerg Med. (2019) 57:791–7. 10.1016/j.jemermed.2019.08.033 31708310

[36] Leon LR Bouchama A. Heat stroke. Compr Physiol. (2015) 5:611–47. 10.1002/cphy.c140017 25880507

[37] Khan AA. Heat related illnesses. Rev Ongoing Challenge. Saudi Med J. (2019) 40:1195–201. 10.15537/smj.2019.12.24727 31828270 PMC6969637

[38] Lian P Braber S Garssen J Wichers HJ Folkerts G Fink-Gremmels J. Beyond heat stress: intestinal integrity disruption and mechanism-based intervention strategies. Nutrients. (2020) 12:3. 10.3390/nu12030734 32168808 PMC7146479

[39] Sertaridou E Papaioannou V Kolios G Pneumatikos I. Gut failure in critical care: old school vs. new school. Ann Gastroenterol. (2015) 28:309–22. 26130136 PMC4480167

[40] Otani S Coopersmith CM. Gut integrity in critical illness. J Intensive Care. (2019) 7:17. 10.1186/s40560-019-0372-6 30923621 PMC6425574

[41] Wirth KJ Scheibenbogen C. Pathophysiology of skeletal muscle disturbances in myalgic encephalomyelitis/chronic fatigue syndrome (ME/CFS). J Transl Med. (2021) 19:162. 10.1186/s12967-021-02833-2 33882940 PMC8058748

[42] Wirth K Scheibenbogen C. A unifying hypothesis of the pathophysiology of myalgic encephalomyelitis/chronic fatigue syndrome (ME/CFS): recognitions from the finding of autoantibodies against ß2-adrenergic receptors. Autoimmun Rev. (2020) 19:102527. 10.1016/j.autrev.2020.102527 32247028

[43] Malato J Sotzny F Bauer S Freitag H Fonseca A Grabowska AD. The SARS-CoV-2 receptor angiotensin-converting enzyme 2 (ACE2) in myalgic encephalomyelitis/chronic fatigue syndrome: a meta-analysis of public DNA methylation and gene expression data. Heliyon. (2021) 7:e07665. 10.1016/j.heliyon.2021.e07665 34341773 PMC8320404

[44] Fluge O Tronstad KJ Mella O. Pathomechanisms and possible interventions in myalgic encephalomyelitis/chronic fatigue syndrome (ME/CFS). J Clin Invest. (2021) 131:14. 10.1172/JCI150377 34263741 PMC8279575

[45] Reynolds GK Lewis DP Richardson AM Lidbury BA. Comorbidity of postural orthostatic tachycardia syndrome and chronic fatigue syndrome in an Australian cohort. J Intern Med. (2014) 275:409–17. 10.1111/joim.12161 24206536

[46] Stewart JM. Chronic orthostatic intolerance and the postural tachycardia syndrome (POTS). J Pediatr. (2004) 145:725–30. 10.1016/j.jpeds.2004.06.084 15580191 PMC4511479

[47] Vernino S Bourne KM Stiles LE Grubb BP Fedorowski A Stewart JM. Postural orthostatic tachycardia syndrome (POTS): State of the science and clinical care from a 2019 national institutes of health expert consensus meeting–part 1. Auton Neurosci. (2021) 235:102828. 10.1016/j.autneu.2021.102828 34144933 PMC8455420

[48] Frith J Zalewski P Klawe JJ Pairman J Bitner A Tafil-Klawe M. Impaired blood pressure variability in chronic fatigue syndrome–a potential biomarker. QJM. (2012) 105:831–8. 10.1093/qjmed/hcs085 22670061

[49] Nelson MJ Bahl JS Buckley JD Thomson RL Davison K. Evidence of altered cardiac autonomic regulation in myalgic encephalomyelitis/chronic fatigue syndrome: a systematic review and meta-analysis. Medicine. (2019) 98:e17600. 10.1097/MD.0000000000017600 31651868 PMC6824690

[50] Armstrong LE Lee EC Armstrong EM. Interactions of gut microbiota, endotoxemia, immune function, and diet in exertional heatstroke. J Sports Med. (2018) 2018:5724575. 10.1155/2018/5724575 29850597 PMC5926483

[51] Hall DM Buettner GR Oberley LW Xu L Matthes RD Gisolfi CV. Mechanisms of circulatory and intestinal barrier dysfunction during whole body hyperthermia. Am J Physiol Heart Circ Physiol. (2001) 280:H509–21. 10.1152/ajpheart.2001.280.2.H509 11158946

[52] Lambert GP. Role of gastrointestinal permeability in exertional heatstroke. Exerc Sport Sci Rev. (2004) 32:185–90. 10.1097/00003677-200410000-00011 15604939

[53] Hifumi T Kondo Y Shimizu K Miyake Y. Heat stroke. J Intensiv Care. (2018) 6:1–8. 10.1186/s40560-018-0298-4 29850022 PMC5964884

[54] Mittal R Coopersmith CM. Redefining the gut as the motor of critical illness. Trends Mol Med. (2014) 20:214–23. 10.1016/j.molmed.2013.08.004 24055446 PMC3959633

[55] Deane A Chapman MJ Fraser RJ Horowitz M. Bench-to-bedside review: the gut as an endocrine organ in the critically ill. Crit Care. (2010) 14:228. 10.1186/cc9039 20887636 PMC3219235

[56] Martinez EE Fasano A Mehta NM. Gastrointestinal function in critical illness—a complex interplay between the nervous and enteroendocrine systems. Pediatric Med. (2020) 3:74. 10.21037/pm-20-74

[57] Soares AD Costa KA Wanner SP Santos RG Fernandes SO Martins FS. Dietary glutamine prevents the loss of intestinal barrier function and attenuates the increase in core body temperature induced by acute heat exposure. Br J Nutr. (2014) 112:1601–10. 10.1017/S0007114514002608 25322775

[58] Singleton KD Wischmeyer PE. Oral glutamine enhances heat shock protein expression and improves survival following hyperthermia. Shock. (2006) 25:295–9. 10.1097/01.shk.0000196548.10634.02 16552363

[59] Cao Y Liu Z Xiao W Gu Z Xiao G Yuan F. 4-Phenylbutyrate prevents endoplasmic reticulum stress-mediated apoptosis induced by heatstroke in the intestines of mice. Shock. (2020) 54:102–9. 10.1097/SHK.0000000000001419 31361709 PMC7289134

[60] Liu CC Chien CH Lin MT. Glucocorticoids reduce interleukin-1 concentration and result in neuroprotective effects in rat heatstroke. J Physiol. (2000) 527:333–43. 10.1111/j.1469-7793.2000.t01-1-00333.x 10970434 PMC2270068

[61] King MA Rollo I Baker LB. Nutritional considerations to counteract gastrointestinal permeability during exertional heat stress. J Appl Physiol. (1985) 130:1754-65. 10.1152/japplphysiol.00072.2021 33955260

[62] Sfera A Osorio C Zapata Martin Del Campo CM Pereida S Maurer S Maldonado JC. Endothelial senescence and chronic fatigue syndrome, a COVID-19 based hypothesis. Front Cell Neurosci. (2021) 15:673217. 10.3389/fncel.2021.673217 34248502 PMC8267916

[63] Maes M Coucke F Leunis JC. Normalization of the increased translocation of endotoxin from gram negative enterobacteria (leaky gut) is accompanied by a remission of chronic fatigue syndrome. Neuro Endocrinol Lett. (2007) 28:739–44. 18063928

[64] Giloteaux L Goodrich JK Walters WA Levine SM Ley RE Hanson MR. Reduced diversity and altered composition of the gut microbiome in individuals with myalgic encephalomyelitis/chronic fatigue syndrome. Microbiome. (2016) 4:30. 10.1186/s40168-016-0171-4 27338587 PMC4918027

[65] Morris G Berk M Carvalho AF Caso JR Sanz Y Maes M. The role of microbiota and intestinal permeability in the pathophysiology of autoimmune and neuroimmune processes with an emphasis on inflammatory bowel disease type 1 diabetes and chronic fatigue syndrome. Curr Pharm Des. (2016) 22:6058–75. 10.2174/1381612822666160914182822 27634186

[66] Morris G Maes M Berk M Puri BK. Myalgic encephalomyelitis or chronic fatigue syndrome: how could the illness develop? Metabolic Brain Disease. (2019) 34:385–415. 10.1007/s11011-019-0388-6 30758706 PMC6428797

[67] Lakhan SE Kirchgessner A. Gut inflammation in chronic fatigue syndrome. Nutr Metab. (2010) 7:79. 10.1186/1743-7075-7-79 20939923 PMC2964729

[68] Maes M Mihaylova I Leunis JC. Increased serum IgA and IgM against LPS of enterobacteria in chronic fatigue syndrome (CFS): indication for the involvement of gram-negative enterobacteria in the etiology of CFS and for the presence of an increased gut-intestinal permeability. J Affect Disord. (2007) 99:237–40. 10.1016/j.jad.2006.08.021 17007934

[69] Zhang ZT Du XM Ma XJ Zong Y Chen JK Yu CL. Activation of the NLRP3 inflammasome in lipopolysaccharide-induced mouse fatigue and its relevance to chronic fatigue syndrome. J Neuroinflammation. (2016) 13:71. 10.1186/s12974-016-0539-1 27048470 PMC4822300

[70] Maes M Leunis JC. Normalization of leaky gut in chronic fatigue syndrome (CFS) is accompanied by a clinical improvement: effects of age, duration of illness and the translocation of LPS from gram-negative bacteria. Neuro Endocrinol Lett. (2008) 29:902–10. 19112401

[71] Anderson G Maes M. Mitochondria and immunity in chronic fatigue syndrome. Prog Neuropsychopharmacol Biol Psychiatry. (2020) 103:109976. 10.1016/j.pnpbp.2020.109976 32470498

[72] Camilleri M Gorman H. Intestinal permeability and irritable bowel syndrome. Neurogastroenterol Motil. (2007) 19:545–52. 10.1111/j.1365-2982.2007.00925.x 17593135

[73] Arrieta MC Bistritz L Meddings JB. Alterations in intestinal permeability. Gut. (2006) 55:1512–20. 10.1136/gut.2005.085373 16966705 PMC1856434

[74] Shing CM Peake JM Lim CL Briskey D Walsh NP Fortes MB. Effects of probiotics supplementation on gastrointestinal permeability, inflammation and exercise performance in the heat. Eur J Appl Physiol. (2014) 114:93–103. 10.1007/s00421-013-2748-y 24150782

[75] Derikx JP Luyer MD Heineman E Buurman WA. Non-invasive markers of gut wall integrity in health and disease. World J Gastroenterol. (2010) 16:5272–9. 10.3748/wjg.v16.i42.5272 21072889 PMC2980675

[76] Sapone A de Magistris L Pietzak M Clemente MG Tripathi A Cucca F. Zonulin upregulation is associated with increased gut permeability in subjects with type 1 diabetes and their relatives. Diabetes. (2006) 55:1443–9. 10.2337/db05-1593 16644703

[77] Fasano A. Leaky gut and autoimmune diseases. Clin Rev Allergy Immunol. (2012) 42:71–8. 10.1007/s12016-011-8291-x 22109896

[78] Mu Q Kirby J Reilly CM Luo XM. Leaky Gut As a Danger Signal for Autoimmune Diseases. Front Immunol. (2017) 8:598. 10.3389/fimmu.2017.00598 28588585 PMC5440529

[79] Collins SM. A role for the gut microbiota in IBS. Nat Rev Gastroenterol Hepatol. (2014) 11:497–505. 10.1038/nrgastro.2014.40 24751910

[80] Service NH. Leaky gut syndrome (2018). Available online at: https://www.heron.nhs.uk/pidd/publicationdetails.aspx?m=1&formatid=24760 (accessed November 1, 2021).

[81] Zhang ZT Gu XL Zhao X He X Shi HW Zhang K. NLRP3 ablation enhances tolerance in heat stroke pathology by inhibiting IL-1beta-mediated neuroinflammation. J Neuroinflammation. (2021) 18:128. 10.1186/s12974-021-02179-y 34092247 PMC8182902

[82] Pedersen BK Febbraio M. Muscle-derived interleukin-6–a possible link between skeletal muscle, adipose tissue, liver, and brain. Brain Behav Immun. (2005) 19:371–6. 10.1016/j.bbi.2005.04.008 15935612

[83] Li P Wang G Zhang XL He GL Luo X Yang J. MicroRNA-155 promotes heat stress-induced inflammation via targeting liver x receptor alpha in Microglia. Front Cell Neurosci. (2019) 13:12. 10.3389/fncel.2019.00012 30778287 PMC6369214

[84] Lin MT Liu HH Yang YL. Involvement of interleukin-1 receptor mechanisms in development of arterial hypotension in rat heatstroke. Am J Physiol. (1997) 273:H2072–7. 10.1152/ajpheart.1997.273.4.H2072 9362278

[85] Lim CL Wilson G Brown L Coombes JS Mackinnon LT. Pre-existing inflammatory state compromises heat tolerance in rats exposed to heat stress. Am J Physiol Regul Integr Comp Physiol. (2007) 292:R186–94. 10.1152/ajpregu.00921.2005 16990481

[86] Chen CM Hou CC Cheng KC Tian RL Chang CP Lin MT. Activated protein C therapy in a rat heat stroke model. Crit Care Med. (2006) 34:1960–6. 10.1097/01.CCM.0000224231.01533.B1 16715032

[87] Yang L Guo H Li Y Meng X Yan L Dan Z. Oleoylethanolamide exerts anti-inflammatory effects on LPS-induced THP-1 cells by enhancing PPARα signaling and inhibiting the NF-κB and ERK1/2/AP-1/STAT3 pathways. Sci Rep. (2016) 6:34611. 10.1038/srep34611 27721381 PMC5056375

[88] Umemura Y Ogura H Matsuura H Ebihara T Shimizu K Shimazu T. Bone marrow-derived mononuclear cell therapy can attenuate systemic inflammation in rat heatstroke. Scand J Trauma Resusc Emerg Med. (2018) 26:97. 10.1186/s13049-018-0566-2 30445981 PMC6240199

[89] Lin X Lin CH Zhao T Zuo D Ye Z Liu L. Quercetin protects against heat stroke-induced myocardial injury in male rats: antioxidative and antiinflammatory mechanisms. Chem Biol Interact. (2017) 265:47–54. 10.1016/j.cbi.2017.01.006 28104348

[90] Chang CY Chen JY Chen SH Cheng TJ Lin MT Hu ML. Therapeutic treatment with ascorbate rescues mice from heat stroke-induced death by attenuating systemic inflammatory response and hypothalamic neuronal damage. Free Radic Biol Med. (2016) 93:84–93. 10.1016/j.freeradbiomed.2015.12.017 26703968

[91] Blomberg J Gottfries CG Elfaitouri A Rizwan M Rosén A. Infection elicited autoimmunity and myalgic encephalomyelitis/chronic fatigue syndrome: an explanatory model. Front Immunol. (2018) 9:229. 10.3389/fimmu.2018.00229 29497420 PMC5818468

[92] Myhill S. Diagnosis and Treatment of Chronic Fatigue Syndrome and Myalgic Encephalitis, 2nd ed.: It's Mitochondria, Not Hypochondria. White River Junction, VT: Chelsea Green Publishing (2018).

[93] Morris G Berk M Galecki P Maes M. The emerging role of autoimmunity in myalgic encephalomyelitis/chronic fatigue syndrome (ME/cfs). Mol Neurobiol. (2014) 49:741–56. 10.1007/s12035-013-8553-0 24068616

[94] Komaroff AL. Inflammation correlates with symptoms in chronic fatigue syndrome. Proc Natl Acad Sci U S A. (2017) 114:8914–6. 10.1073/pnas.1712475114 28811366 PMC5576849

[95] Sweetman E Noble A Edgar C Mackay A Helliwell A Vallings R. Current research provides insight into the biological basis and diagnostic potential for myalgic encephalomyelitis/chronic fatigue syndrome (ME/CFS). Diagnostics. (2019) 9:73. 10.3390/diagnostics9030073 31295930 PMC6787691

[96] Glassford JA. The neuroinflammatory etiopathology of myalgic encephalomyelitis/chronic fatigue syndrome (ME/CFS). Front Physiol. (2017) 8:88. 10.3389/fphys.2017.00088 28261110 PMC5314655

[97] Tomas C Newton J. Metabolic abnormalities in chronic fatigue syndrome/myalgic encephalomyelitis: a mini-review. Biochem Soc Trans. (2018) 46:547–53. 10.1042/BST20170503 29666214

[98] Maes M Kubera M Uytterhoeven M Vrydags N Bosmans E. Increased plasma peroxides as a marker of oxidative stress in myalgic encephalomyelitis/chronic fatigue syndrome (ME/CFS). Med Sci Monit. (2011) 17:4. 10.12659/MSM.881699 21455120 PMC3539515

[99] Montoya JG Holmes TH Anderson JN Maecker HT Rosenberg-Hasson Y Valencia IJ. Cytokine signature associated with disease severity in chronic fatigue syndrome patients. Proc Natl Acad Sci USA. (2017) 114:E7150–E8. 10.1073/pnas.1710519114 28760971 PMC5576836

[100] Hornig M Montoya JG Klimas NG Levine S Felsenstein D Bateman L. Distinct plasma immune signatures in ME/CFS are present early in the course of illness. Sci Adv. (2015) 1:e1400121. 10.1126/sciadv.1400121 26079000 PMC4465185

[101] Paul BD Lemle MD Komaroff AL Snyder SH. Redox imbalance links COVID-19 and myalgic encephalomyelitis/chronic fatigue syndrome. Proc Natl Acad Sci U S A. (2021) 118:34. 10.1073/pnas.2024358118 34400495 PMC8403932

[102] Armstrong CW McGregor NR Lewis DP Butt HL Gooley PR. Metabolic profiling reveals anomalous energy metabolism and oxidative stress pathways in chronic fatigue syndrome patients. Metabolomics. (2015) 11:1626–39. 10.1007/s11306-015-0816-5

[103] Morris G Maes M. Mitochondrial dysfunctions in myalgic encephalomyelitis/chronic fatigue syndrome explained by activated immuno-inflammatory, oxidative and nitrosative stress pathways. Metab Brain Dis. (2014) 29:19–36. 10.1007/s11011-013-9435-x 24557875

[104] Hatziagelaki E Adamaki M Tsilioni I Dimitriadis G Theoharides TC. Myalgic encephalomyelitis/chronic fatigue syndrome-metabolic disease or disturbed homeostasis due to focal inflammation in the hypothalamus? J Pharmacol Exp Ther. (2018) 367:155–67. 10.1124/jpet.118.250845 30076265

[105] Morris G Anderson G Maes M. Hypothalamic-Pituitary-Adrenal Hypofunction in myalgic encephalomyelitis (me)/chronic fatigue syndrome (cfs) as a consequence of activated immune-inflammatory and oxidative and nitrosative pathways. Mol Neurobiol. (2017) 54:6806–19. 10.1007/s12035-016-0170-2 27766535

[106] Pall M. The NO/ONOO-cycle mechanism as the cause of chronic fatigue syndrome/myalgia encephalomyelitis In: Svoboda E Zelenjcik K editors. Chronic Fatigue Syndrome: Symptoms, Causes and Prevention, London: Nova Publishers (2009).

[107] Morris G Maes M. Oxidative and nitrosative stress and immune-inflammatory pathways in patients with myalgic encephalomyelitis (me)/chronic fatigue syndrome (CFS). Curr Neuropharmacol. (2014) 12:168–85. 10.2174/1570159X11666131120224653 24669210 PMC3964747

[108] Shibolet S Coll R Gilat T Sohar E. Heatstroke: its clinical picture and mechanism in 36 cases. Q J Med. (1967) 36:525–48. 6077230

[109] Chao TC Sinniah R Pakiam JE. Acute heat stroke deaths. Pathology. (1981) 13:145–56. 10.3109/00313028109086837 7220095

[110] Lin MT. Pathogenesis of an experimental heatstroke model. Clin Exp Pharmacol Physiol. (1999) 26:826–7. 10.1046/j.1440-1681.1999.03137.x 10549412

[111] Yamaguchi T Shimizu K Kokubu Y Nishijima M Takeda S Ogura H. Effect of heat stress on blood-brain barrier integrity in iPS cell-derived microvascular endothelial cell models. PLoS ONE. (2019) 14:e0222113. 10.1371/journal.pone.0222113 31483843 PMC6726235

[112] Nakatomi Y Kuratsune H Watanabe Y. Neuroinflammation in the brain of patients with myalgic encephalomyelitis/chronic fatigue syndrome. Brain Nerve. (2018) 70:19–25. 10.11477/mf.1416200945 29348371

[113] Nakatomi Y Mizuno K Ishii A Wada Y Tanaka M Tazawa S. Neuroinflammation in patients with chronic fatigue syndrome/myalgic encephalomyelitis: an (1)(1)C-(R)-PK11195 PET study. J Nucl Med. (2014) 55:945–50. 10.2967/jnumed.113.131045 24665088

[114] Mueller C Lin JC Sheriff S Maudsley AA Younger JW. Evidence of widespread metabolite abnormalities in Myalgic encephalomyelitis/chronic fatigue syndrome: assessment with whole-brain magnetic resonance spectroscopy. Brain Imaging Behav. (2020) 14:562–72. 10.1007/s11682-018-0029-4 30617782 PMC6612467

[115] Zeineh MM Kang J Atlas SW Raman MM Reiss AL Norris JL. Right arcuate fasciculus abnormality in chronic fatigue syndrome. Radiology. (2015) 274:517–26. 10.1148/radiol.14141079 25353054

[116] Ferrero K Silver M Cocchetto A Masliah E Langford D. CNS findings in chronic fatigue syndrome and a neuropathological case report. J Investig Med. (2017) 65:974–83. 10.1136/jim-2016-000390 28386034

[117] van Campen CMC Verheugt FWA Rowe PC Visser FC. Cerebral blood flow is reduced in ME/CFS during head-up tilt testing even in the absence of hypotension or tachycardia: a quantitative, controlled study using doppler echography. Clinical Neurophysiology Practice. (2020) 5:50–8. 10.1016/j.cnp.2020.01.003 32140630 PMC7044650

[118] Bragee B Michos A Drum B Fahlgren M Szulkin R Bertilson BC. Signs of intracranial hypertension, hypermobility, and craniocervical obstructions in patients with myalgic encephalomyelitis/chronic fatigue syndrome. Front Neurol. (2020) 11:828. 10.3389/fneur.2020.00828 32982905 PMC7485557

[119] Hulens M Rasschaert R Vansant G Stalmans I Bruyninckx F Dankaerts W. The link between idiopathic intracranial hypertension, fibromyalgia, and chronic fatigue syndrome: exploration of a shared pathophysiology. J Pain Res. (2018) 11:3129–40. 10.2147/JPR.S186878 30573989 PMC6292399

[120] Louveau A Harris TH Kipnis J. Revisiting the mechanisms of CNS immune privilege. Trends Immunol. (2015) 36:569–77. 10.1016/j.it.2015.08.006 26431936 PMC4593064

[121] Forrester JV McMenamin PG Dando SJ. CNS infection and immune privilege. Nat Rev Neurosci. (2018) 19:655–71. 10.1038/s41583-018-0070-8 30310148

[122] Chen HS Tong HS Zhao Y Hong CY Bin JP Su L. Differential expression pattern of exosome long non-coding rnas (lncRNAs) and micrornas (mirnas) in vascular endothelial cells under heat stroke. Med Sci Monit. (2018) 24:7965–74. 10.12659/MSM.909983 30399613 PMC6234752

[123] Bouchama A Roberts G Al Mohanna F El-Sayed R Lach B Chollet-Martin S. Inflammatory, hemostatic, and clinical changes in a baboon experimental model for heatstroke. J Appl Physiol. (1985) 98:697–705. 10.1152/japplphysiol.00461.2004 15475604

[124] Matsumoto H Takeba J Umakoshi K Nakabayashi Y Moriyama N Annen S. Successful treatment for disseminated intravascular coagulation (DIC) corresponding to phenotype changes in a heat stroke patient. J Intensive Care. (2019) 7:2. 10.1186/s40560-019-0359-3 30675362 PMC6332900

[125] Kobayashi K Mimuro S Sato T Kobayashi A Kawashima S Makino H. Dexmedetomidine preserves the endothelial glycocalyx and improves survival in a rat heatstroke model. J Anesth. (2018) 32:880–5. 10.1007/s00540-018-2568-7 30374889

[126] Berg D Berg LH Couvaras J Harrison H. Chronic fatigue syndrome and/or fibromyalgia as a variation of antiphospholipid antibody syndrome: an explanatory model and approach to laboratory diagnosis. Blood Coagul Fibrinol. (1999) 10:435–8. 10.1097/00001721-199910000-00006 10695770

[127] Hannan KL Berg DE Baumzweiger W Harrison HH Berg LH Ramirez R. Activation of the coagulation system in Gulf War Illness: a potential pathophysiologic link with chronic fatigue syndrome. a laboratory approach to diagnosis. Blood Coagul Fibrinol. (2000) 11:673–8. 10.1097/00001721-200010000-00013 11085289

[128] Brewer JH Berg D. Hypercoaguable state associated with active human herpesvirus-6 (HHV-6) viremia in patients with chronic fatigue syndrome. J Chronic Fatigue Syndrome. (2001) 8:111–6. 10.1300/J092v08n03_10

[129] Kennedy G Norris G Spence V McLaren M Belch JJ. Is chronic fatigue syndrome associated with platelet activation? Blood Coagul Fibrinol. (2006) 17:89–92. 10.1097/01.mbc.0000214705.80997.73 16479189

[130] Newton DJ Kennedy G Chan KK Lang CC Belch JJ Khan F. Large and small artery endothelial dysfunction in chronic fatigue syndrome. Int J Cardiol. (2012) 154:335–6. 10.1016/j.ijcard.2011.10.030 22078396

[131] Sorland K Sandvik MK Rekeland IG Ribu L Smastuen MC Mella O. Reduced endothelial function in myalgic encephalomyelitis/chronic fatigue syndrome-results from open-label cyclophosphamide intervention study. Front Med. (2021) 8:642710. 10.3389/fmed.2021.642710 33829023 PMC8019750

[132] Blauensteiner J Bertinat R Leon LE Riederer M Sepulveda N Westermeier F. Altered endothelial dysfunction-related miRs in plasma from ME/CFS patients. Sci Rep. (2021) 11:10604. 10.1038/s41598-021-89834-9 34011981 PMC8134566

[133] Li L Su Z Zou Z Tan H Cai D Su L. Ser46 phosphorylation of p53 is an essential event in prolyl-isomerase Pin1-mediated p53-independent apoptosis in response to heat stress. Cell Death Dis. (2019) 10:96. 10.1038/s41419-019-1316-8 30718466 PMC6362080

[134] Zhang Y Wang S Wang X Zan Q Yu X Fan L. Monitoring of the decreased mitochondrial viscosity during heat stroke with a mitochondrial AIE probe. Anal Bioanal Chem. (2021) 413:3823–31. 10.1007/s00216-021-03335-2 33934190

[135] Schreiner P Harrer T Scheibenbogen C Lamer S Schlosser A Naviaux RK. Human herpesvirus-6 reactivation, mitochondrial fragmentation, and the coordination of antiviral and metabolic phenotypes in myalgic encephalomyelitis/chronic fatigue syndrome. Immunohorizons. (2020) 4:201–15. 10.4049/immunohorizons.2000006 32327453

[136] Birdwell CE West P. Exploring a role for Human Herpesvirus 6B-mediated mitochondrial dysfunction in dysregulating antiviral innate immunity. The Journal of Immunology. (2018) 200:50.

[137] Keller BA Pryor JL Giloteaux L. Inability of myalgic encephalomyelitis/chronic fatigue syndrome patients to reproduce VO(2)peak indicates functional impairment. J Transl Med. (2014) 12:104. 10.1186/1479-5876-12-104 24755065 PMC4004422

[138] Myhill S Booth NE McLaren-Howard J. Chronic fatigue syndrome and mitochondrial dysfunction. Int J Clin Exp Med. (2009) 2:1–16. 19436827 PMC2680051

[139] Esfandyarpour R Kashi A Nemat-Gorgani M Wilhelmy J Davis RW. A nanoelectronics-blood-based diagnostic biomarker for myalgic encephalomyelitis/chronic fatigue syndrome (ME/CFS). Proc Natl Acad Sci U S A. (2019) 116:10250–7. 10.1073/pnas.1901274116 31036648 PMC6535016

[140] Vernon SD Whistler T Cameron B Hickie IB Reeves WC Lloyd A. Preliminary evidence of mitochondrial dysfunction associated with post-infective fatigue after acute infection with Epstein Barr virus. BMC Infect Dis. (2006) 6:15. 10.1186/1471-2334-6-15 16448567 PMC1373655

[141] Fluge O Mella O Bruland O Risa K Dyrstad SE Alme K. Metabolic profiling indicates impaired pyruvate dehydrogenase function in myalgic encephalopathy/chronic fatigue syndrome. JCI Insight. (2016) 1:e89376. 10.1172/jci.insight.89376 28018972 PMC5161229

[142] Germain A Ruppert D Levine SM Hanson MR. Metabolic profiling of a myalgic encephalomyelitis/chronic fatigue syndrome discovery cohort reveals disturbances in fatty acid and lipid metabolism. Mol Biosyst. (2017) 13:371–9. 10.1039/C6MB00600K 28059425 PMC5365380

[143] Naviaux RK Naviaux JC Li K Bright AT Alaynick WA Wang L. Metabolic features of chronic fatigue syndrome. Proc Natl Acad Sci U S A. (2016) 113:E5472–80. 10.1073/pnas.1607571113 27573827 PMC5027464

[144] Naviaux RK. Perspective: Cell danger response Biology—The new science that connects environmental health with mitochondria and the rising tide of chronic illness. Mitochondrion. (2020) 51:40–5. 10.1016/j.mito.2019.12.005 31877376

[145] Naviaux RK. Metabolic features and regulation of the healing cycle-A new model for chronic disease pathogenesis and treatment. Mitochondrion. (2019) 46:278–97. 10.1016/j.mito.2018.08.001 30099222

[146] Bouchama A Aziz MA Mahri SA Gabere MN Dlamy MA Mohammad S. A model of exposure to extreme environmental heat uncovers the human transcriptome to heat stress. Sci Rep. (2017) 7:9429. 10.1038/s41598-017-09819-5 28842615 PMC5573409

[147] Kitami T Fukuda S Kato T Yamaguti K Nakatomi Y Yamano E. Deep phenotyping of myalgic encephalomyelitis/chronic fatigue syndrome in Japanese population. Sci Rep. (2020) 10:19933. 10.1038/s41598-020-77105-y 33199820 PMC7669873

[148] Frampton D Kerr J Harrison TJ Kellam P. Assessment of a 44 gene classifier for the evaluation of chronic fatigue syndrome from peripheral blood mononuclear cell gene expression. PLoS One. (2011) 6:e16872. 10.1371/journal.pone.0016872 21479222 PMC3068152

[149] Gow JW Hagan S Herzyk P Cannon C Behan PO Chaudhuri A. A gene signature for post-infectious chronic fatigue syndrome. BMC Med Genomics. (2009) 2:38. 10.1186/1755-8794-2-38 19555476 PMC2716361

[150] Vernon SD Unger ER Dimulescu IM Rajeevan M Reeves WC. Utility of the blood for gene expression profiling and biomarker discovery in chronic fatigue syndrome. Dis Markers. (2002) 18:193–9. 10.1155/2002/892374 12590173 PMC3851413

[151] Nguyen CB Alsoe L Lindvall JM Sulheim D Fagermoen E Winger A. Whole blood gene expression in adolescent chronic fatigue syndrome: an exploratory cross-sectional study suggesting altered B cell differentiation and survival. J Transl Med. (2017) 15:102. 10.1186/s12967-017-1201-0 28494812 PMC5426002

[152] Kerr JR Petty R Burke B Gough J Fear D Sinclair LI. Gene expression subtypes in patients with chronic fatigue syndrome/myalgic encephalomyelitis. J Infect Dis. (2008) 197:1171–84. 10.1086/533453 18462164

[153] Sweetman E Ryan M Edgar C MacKay A Vallings R Tate W. Changes in the transcriptome of circulating immune cells of a New Zealand cohort with myalgic encephalomyelitis/chronic fatigue syndrome. Int J Immunopathol Pharmacol. (2019) 33:2058738418820402. 10.1177/2058738418820402 30791746 PMC6350121

[154] Saiki T Kawai T Morita K Ohta M Saito T Rokutan K. Identification of marker genes for differential diagnosis of chronic fatigue syndrome. Mol Med. (2008) 14:599–607. 10.2119/2007-00059.Saiki 18596870 PMC2442021

[155] Nepotchatykh E Elremaly W Caraus I Godbout C Leveau C Chalder L. Profile of circulating microRNAs in myalgic encephalomyelitis and their relation to symptom severity, and disease pathophysiology. Scientific Reports. (2020) 10:19620. 10.1038/s41598-020-76438-y 33184353 PMC7665057

[156] Epstein Y. Heat intolerance: predisposing factor or residual injury? Med Sci Sports Exerc. (1990) 22:29–35. 10.1249/00005768-199002000-00006 2406544

[157] Armstrong LE De Luca JP Hubbard RW. Time course of recovery and heat acclimation ability of prior exertional heatstroke patients. Med Sci Sports Exerc. (1990) 22:36–48. 10.1249/00005768-199002000-00007 2406545

[158] Lim CL. Fundamental concepts of human thermoregulation and adaptation to heat: a review in the context of global warming. Int J Environ Res Public Health. (2020) 17:21. 10.3390/ijerph17217795 33114437 PMC7662600

[159] Nieman DC Pedersen BK. Exercise and immune function. Sports Med. (1999) 27:73–80. 10.2165/00007256-199927020-00001 10091272

[160] Lakier Smith L. Overtraining, excessive exercise, and altered immunity: is this a T helper-1 vs. T helper-2 lymphocyte response? Sports Med. (2003) 33:347–64. 10.2165/00007256-200333050-00002 12696983

[161] Gifford RM Todisco T Stacey M Fujisawa T Allerhand M Woods DR. Risk of heat illness in men and women: a systematic review and meta-analysis. Environ Res. (2019) 171:24–35. 10.1016/j.envres.2018.10.020 30641370

[162] Kazman JB Purvis DL Heled Y Lisman P Atias D Van Arsdale S. Women and exertional heat illness: identification of gender specific risk factors. US Army Med Dep J. (2015) :58-66. 26101907

[163] Alele F Malau-Aduli B Malau-Aduli A Crowe M. Systematic review of gender differences in the epidemiology and risk factors of exertional heat illness and heat tolerance in the armed forces. BMJ Open. (2020) 10:e031825. 10.1136/bmjopen-2019-031825 32265238 PMC7245403

[164] Moseley PL. Heat shock proteins and the inflammatory response. Ann N Y Acad Sci. (1998) 856:206–13. 10.1111/j.1749-6632.1998.tb08327.x 9917879

[165] Moseley PL. Heat shock proteins and heat adaptation of the whole organism. J Appl Physiol. (1985) 83:1413–7. 10.1152/jappl.1997.83.5.1413 9375300

[166] Shen HH Tseng YS Kuo NC Kung CW Amin S Lam KK. Alpha-Lipoic acid protects cardiomyocytes against heat stroke-induced apoptosis and inflammatory responses associated with the induction of hsp70 and activation of autophagy. Mediators Inflamm. (2019) 2019:8187529. 10.1155/2019/8187529 31885498 PMC6914879

[167] Lacerda EM Geraghty K Kingdon CC Palla L Nacul L. A logistic regression analysis of risk factors in ME/CFS pathogenesis. BMC Neurol. (2019) 19:275. 10.1186/s12883-019-1468-2 31699051 PMC6839177

[168] Buchwald D Cheney PR Peterson DL Henry B Wormsley SB Geiger A. A chronic illness characterized by fatigue, neurologic and immunologic disorders, and active human herpesvirus type 6 infection. Ann Intern Med. (1992) 116:103–13. 10.7326/0003-4819-116-2-103 1309285

[169] Ablashi DV. Viral studies of chronic fatigue syndrome. Clin Infect Dis. (1994) 18:S130–3. 10.1093/clinids/18.Supplement_1.S130 8148440

[170] Rasa S Nora-Krukle Z Henning N Eliassen E Shikova E Harrer T. Chronic viral infections in myalgic encephalomyelitis/chronic fatigue syndrome (ME/CFS). J Transl Med. (2018) 16:268. 10.1186/s12967-018-1644-y 30285773 PMC6167797

[171] Kerr JR. Epstein-Barr virus induced gene-2 upregulation identifies a particular subtype of chronic fatigue syndrome/myalgic encephalomyelitis. Front Pediatr. (2019) 7:59. 10.3389/fped.2019.00059 30918887 PMC6424879

[172] Nater UM Maloney E Heim C Reeves WC. Cumulative life stress in chronic fatigue syndrome. Psychiatry Res. (2011) 189:318–20. 10.1016/j.psychres.2011.07.015 21840607

[173] Gaab J Rohleder N Heitz V Engert V Schad T Schurmeyer TH. Stress-induced changes in LPS-induced pro-inflammatory cytokine production in chronic fatigue syndrome. Psychoneuroendocrinology. (2005) 30:188–98. 10.1016/j.psyneuen.2004.06.008 15471616

[174] Lupo GFD Rocchetti G Lucini L Lorusso L Manara E Bertelli M. Potential role of microbiome in chronic fatigue syndrome/myalgic encephalomyelits (CFS/ME). Sci Rep. (2021) 11:7043. 10.1038/s41598-021-86425-6 33782445 PMC8007739

[175] Frémont M Coomans D Massart S De Meirleir K. High-throughput 16S rRNA gene sequencing reveals alterations of intestinal microbiota in myalgic encephalomyelitis/chronic fatigue syndrome patients. Anaerobe. (2013) 22:50–6. 10.1016/j.anaerobe.2013.06.002 23791918

[176] Sheedy JR Wettenhall RE Scanlon D Gooley PR Lewis DP McGregor N. Increased d-lactic acid intestinal bacteria in patients with chronic fatigue syndrome. In Vivo. (2009) 23:621–8. 19567398

[177] Tsai SY Chen HJ Lio CF Kuo CF Kao AC Wang WS. Increased risk of chronic fatigue syndrome in patients with inflammatory bowel disease: a population-based retrospective cohort study. J Transl Med. (2019) 17:55. 10.1186/s12967-019-1797-3 30795765 PMC6387539

[178] Safadi JM Quinton AMG Lennox BR Burnet PWJ Minichino A. Gut dysbiosis in severe mental illness and chronic fatigue: a novel trans-diagnostic construct? a systematic review and meta-analysis. Mol Psychiatry. (2021) 21:1032. 10.1038/s41380-021-01032-1 33558650 PMC8960409

[179] Ghali A Richa P Lacout C Gury A Beucher AB Homedan C. Epidemiological and clinical factors associated with post-exertional malaise severity in patients with myalgic encephalomyelitis/chronic fatigue syndrome. J Transl Med. (2020) 18:246. 10.1186/s12967-020-02419-4 32571354 PMC7309998

[180] Jammes Y Steinberg JG Delliaux S Brégeon F. Chronic fatigue syndrome combines increased exercise-induced oxidative stress and reduced cytokine and Hsp responses. J Intern Med. (2009) 266:196–206. 10.1111/j.1365-2796.2009.02079.x 19457057

[181] Gerwyn M Maes M. Mechanisms explaining muscle fatigue and muscle pain in patients with myalgic encephalomyelitis/chronic fatigue syndrome (ME/CFS): a review of recent findings. Curr Rheumatol Rep. (2017) 19:1. 10.1007/s11926-017-0628-x 28116577

[182] Jammes Y Steinberg JG Delliaux S. Chronic fatigue syndrome: acute infection and history of physical activity affect resting levels and response to exercise of plasma oxidant/antioxidant status and heat shock proteins. J Intern Med. (2012) 272:74–84. 10.1111/j.1365-2796.2011.02488.x 22112145

[183] Jammes Y Retornaz F. Understanding neuromuscular disorders in chronic fatigue syndrome. F1000Res. (2019) 8. 10.12688/f1000research.18660.1 31814961 PMC6883394

[184] Perez M Jaundoo R Hilton K Del Alamo A Gemayel K Klimas NG. Genetic predisposition for immune system, hormone, and metabolic dysfunction in myalgic encephalomyelitis/chronic fatigue syndrome: a pilot study. Front Pediatric. (2019) 7:206. 10.3389/fped.2019.00206 31179255 PMC6542994

[185] Carlo-Stella N Badulli C De Silvestri A Bazzichi L Martinetti M Lorusso L. A first study of cytokine genomic polymorphisms in CFS: positive association of TNF-857 and IFNgamma 874 rare alleles. Clin Exp Rheumatol. (2006) 24:179-82. 16762155

[186] Vangeel E Van Den Eede F Hompes T Izzi B Del Favero J Moorkens G. chronic fatigue syndrome and dna hypomethylation of the glucocorticoid receptor gene promoter 1f region: associations with hpa axis hypofunction and childhood trauma. Psychosom Med. (2015) 77:853–62. 10.1097/PSY.0000000000000224 26230484

[187] Kashi AA Davis RW Phair RD. The IDO metabolic trap hypothesis for the etiology of ME/CFS. Diagnostics. (2019) 9:82. 10.3390/diagnostics9030082 31357483 PMC6787624

[188] Dibble JJ McGrath SJ Ponting CP. Genetic risk factors of ME/CFS: a critical review. Hum Mol Genet. (2020) 29(R1):R117–R24. 10.1093/hmg/ddaa169 32744306 PMC7530519

[189] Grabowska AD Lacerda EM Nacul L Sepulveda N. Review of the quality control checks performed by current genome-wide and targeted-genome association studies on myalgic encephalomyelitis/chronic fatigue syndrome. Front Pediatr. (2020) 8:293. 10.3389/fped.2020.00293 32596192 PMC7304330

[190] Kirby TO Ochoa-Reparaz J. The gut microbiome in multiple sclerosis: a potential therapeutic avenue. Med Sci. (2018) 6:3. 10.3390/medsci6030069 30149548 PMC6163724

[191] Taneja V. Arthritis susceptibility and the gut microbiome. FEBS Lett. (2014) 588:4244–9. 10.1016/j.febslet.2014.05.034 24873878 PMC4246018

[192] Erdrich S Hawrelak JA Myers SP Harnett JE. Determining the association between fibromyalgia, the gut microbiome and its biomarkers: a systematic review. BMC Musculoskelet Disord. (2020) 21:181–9. 10.1186/s12891-020-03201-9 32192466 PMC7083062

[193] Küçükemre Aydin B Yildiz M Akgün A Topal N Adal E Önal H. Children with hashimoto's thyroiditis have increased intestinal permeability: results of a pilot study. J Clin Res Pediatr Endocrinol. (2020) 12:303–7. 10.4274/jcrpe.galenos.2020.2019.0186 31990165 PMC7499128

[194] Morris G Maes M. Myalgic encephalomyelitis/chronic fatigue syndrome and encephalomyelitis disseminata/multiple sclerosis show remarkable levels of similarity in phenomenology and neuroimmune characteristics. BMC Med. (2013) 11:205. 10.1186/1741-7015-11-205 24229326 PMC3847236

[195] Maes M Twisk FNM Kubera M Ringel K. Evidence for inflammation and activation of cell-mediated immunity in myalgic encephalomyelitis/chronic fatigue syndrome (ME/CFS): increased interleukin-1, tumor necrosis factor-α, PMN-elastase, lysozyme and neopterin. J Affect Disord. (2012) 136:933–9. 10.1016/j.jad.2011.09.004 21975140

[196] Natelson BH. Myalgic encephalomyelitis/chronic fatigue syndrome and fibromyalgia: definitions, similarities, and differences. Clin Ther. (2019) 41:612–8. 10.1016/j.clinthera.2018.12.016 30795933 PMC6589349

[197] Sotzny F Blanco J Capelli E Castro-Marrero J Steiner S Murovska M. Myalgic encephalomyelitis/chronic fatigue syndrome—evidence for an autoimmune disease. Autoimmunity Reviews. (2018) 17:601–9. 10.1016/j.autrev.2018.01.009 29635081

[198] Castro-Marrero J Faro M Aliste L Sáez-Francàs N Calvo N Martínez-Martínez A. Comorbidity in chronic fatigue syndrome/myalgic encephalomyelitis: a nationwide population-based cohort study. Psychosomatics. (2017) 58:533–43. 10.1016/j.psym.2017.04.010 28596045

[199] Rawal G Yadav S Kumar R. Post-intensive care syndrome: an overview. J Transl Int Med. (2017) 5:90–2. 10.1515/jtim-2016-0016 28721340 PMC5506407

[200] Smith S Rahman O. Post intensive care syndrome [Updated 2020 Jun 25]In: StatPearls [Internet]. Treasure Island (FL): StatPearls Publishing. (2020).

[201] Van Aerde N Van Dyck L Vanhorebeek I Van den Berghe G. sEndocrinopathy of the critically Ill, In: Preiser J-C Herridge M Azoulay E editors. Post-Intensive Care Syndrome. Cham: Springer International Publishing. (2020), 125–43.

[202] Van den Berghe GH. Acute and prolonged critical illness are two distinct neuroendocrine paradigms. Verh K Acad Geneeskd Belg. (1998) 60:487–518; discussion −20. 10.1210/jc.83.6.1827 10230322

[203] Vanhorebeek I Van den Berghe G. The neuroendocrine response to critical illness is a dynamic process. Crit Care Clin. (2006) 22:1–15. 10.1016/j.ccc.2005.09.004 16399016

[204] Van den Berghe G. On the neuroendocrinopathy of critical illness. perspectives for feeding and novel treatments. Am J Respir Crit Care Med. (2016) 194:1337–48. 10.1164/rccm.201607-1516CI 27611700

[205] Mancini A Di Segni C Raimondo S Olivieri G Silvestrini A Meucci E. Thyroid hormones, oxidative stress, and inflammation. Mediators Inflamm. (2016) 2016:6757154. 10.1155/2016/6757154 27051079 PMC4802023

[206] Chatzitomaris A Hoermann R Midgley JE Hering S Urban A Dietrich B. Thyroid allostasis–adaptive responses of thyrotropic feedback control to conditions of strain, stress, and developmental programming. Front Endocrinol. (2017) 8:163. 10.3389/fendo.2017.00163 28775711 PMC5517413

[207] Greenhalgh T Knight M A'Court C Buxton M Husain L. Management of post-acute covid-19 in primary care. BMJ. (2020) 370:m3026. 10.1136/bmj.m3026 32784198

[208] Dani M Dirksen A Taraborrelli P Torocastro M Panagopoulos D Sutton R. Autonomic dysfunction in “long COVID”: rationale, physiology and management strategies. Clin Med. (2020) 20:. 10.7861/clinmed.2020-0896 33243837 PMC7850225

[209] Huang C Huang L Wang Y Li X Ren L Gu X. 6-month consequences of COVID-19 in patients discharged from hospital: a cohort study. The Lancet. (2021) 397:220–32. 10.1016/S0140-6736(20)32656-8 33428867 PMC7833295

[210] Townsend L Dyer AH Jones K Dunne J Mooney A Gaffney F. Persistent fatigue following SARS-CoV-2 infection is common and independent of severity of initial infection. PLOS ONE. (2020) 15:e0240784. 10.1371/journal.pone.0240784 33166287 PMC7652254

[211] Komaroff AL Bateman L. Will COVID-19 lead to myalgic encephalomyelitis/chronic fatigue syndrome? Front Med. (2021) 7 (1132). 10.3389/fmed.2020.606824 33537329 PMC7848220

[212] Wildwing T Holt N. The neurological symptoms of COVID-19: a systematic overview of systematic reviews, comparison with other neurological conditions and implications for healthcare services. Therapeutic Adv Chronic Dis. (2021) 12:2040622320976979. 10.1177/2040622320976979 33796241 PMC7970685

[213] Proal AD VanElzakker MB. Long COVID or post-acute sequelae of Covid-19 (pasc): an overview of biological factors that may contribute to persistent symptoms. Front Microbiol. (2021) 12:698169. 10.3389/fmicb.2021.698169 34248921 PMC8260991

[214] Mackay A. A paradigm for post-Covid-19 fatigue syndrome analogous to ME/CFS. Front Neurol. (2021) 12:701419. 10.3389/fneur.2021.701419 34408721 PMC8365156

[215] Komaroff AL Lipkin WI. Insights from myalgic encephalomyelitis/chronic fatigue syndrome may help unravel the pathogenesis of postacute COVID-19 syndrome. Trends Mol Med. (2021) 27:895–906. 10.1016/j.molmed.2021.06.002 34175230 PMC8180841

[216] Comella PH Gonzalez-Kozlova E Kosoy R Charney AW Peradejordi IF Chandrasekar S. A molecular network approach reveals shared cellular and molecular signatures between chronic fatigue syndrome and other fatiguing illnesses. [Preprint] medRxiv. (2021). Available online at: https://www.medrxiv.org/content/10.1101/2021.01.29.21250755v1 (accessed October 5, 2021).

